# Antagonistic interaction between jasmonic acid and cytokinin in xylem development

**DOI:** 10.1038/s41598-017-10634-1

**Published:** 2017-08-31

**Authors:** Geupil Jang, Sun Hyun Chang, Tae Young Um, Sangyool Lee, Ju-Kon Kim, Yang Do Choi

**Affiliations:** 1 Department of Agricultural Biotechnology and Research Institute of Agriculture and Life Sciences, Seoul National University, Seoul, 151-921 Korea; 2 Graduate School of International Agricultural Technology and Crop Biotechnology Institute/Green BioScience and Technology, Seoul National University, Pyeongchang, 232-916 Korea

## Abstract

Developmental flexibility under stress conditions largely relies on the interactions between hormones that mediate stress responses and developmental processes. In this study, we showed that the stress hormone jasmonic acid (JA) induces formation of extra xylem in the roots of wild-type *Arabidopsis thaliana* (Col-0). JA signaling mutants such as *coronatine insensitive1-1* and *jasmonate resistant1-1* did not form extra xylem in response to JA, but the JA biosynthesis mutant *oxophytodienoate-reductase3* did form extra xylem. These observations suggested that the JA response promotes xylem development. To understand the mechanism, we examined the regulatory interaction between JA and cytokinin, a negative regulator of xylem development. JA treatment reduced cytokinin responses in the vasculature, and exogenous cytokinin nullified the effect of JA on formation of extra xylem. A time-course experiment showed that suppression of cytokinin responses by JA does not occur rapidly, but the JA-mediated xylem phenotype is tightly linked to the suppression of the cytokinin response. Further analysis of *arabidopsis histidine phosphotransfer protein6-1* and *myc2-3* mutants revealed that the JA-responsive transcription factor MYC2 regulates the expression of *AHP6* in response to JA and expression of *AHP6* is involved in the JA-mediated xylem phenotype.

## Introduction

Molecular and genetic studies have identified many phytohormones and have shown that the activities of these hormones largely overlap, although each hormone has specific signaling pathways that act non-redundantly. These findings suggest that the interplay between phytohormones dynamically regulates plant development and physiology^1^. For example, cytokinin interacts with auxin in the regulation of all aspects of plant development^2, 3^.

JA regulates plant responses to abiotic and biotic stresses and modulates plant development, including stamen filament growth, root growth, and senescence^4–8^. JA is biosynthesized from linolenic acid via the octadecanoid pathway, and then further metabolized to a JA-isoleucine conjugate (JA-Ile)^9, 10^. The interaction between JA-Ile and the CORONATINE INSENSITIVE1 (COI1) receptor provokes proteolysis of transcriptional repressor JASMONATE ZIM-DOMAIN (JAZ) proteins, and the degradation of JAZs leads to release of the MYC2 transcription factor^11–13^. MYC2 regulates JA responses by controlling the expression of JA-responsive genes and plays an essential role in modulating plant defense and development in response to JA. For example, *myc2* mutant plants exhibit enhanced resistance to pathogens such as *Pseudomonas syringae* pv*. tomato* DC3000, *Botrytis cinerea*, and *Fusarium oxysporum* compared to wild-type plants, and JA-mediated inhibition of root growth is suppressed in *myc2* mutant plants^14, 15^. These observations suggest that MYC2 regulates the expression of key genes responsible for the modulation of defense and development in response to JA.

Previous studies showed that crosstalk between JA and other hormones modulates plant defense and development. JA interacts with ethylene for defense against necrotrophic fungi and herbivorous insects or for development of apical hook^16–18^. JA interacts with gibberellic acid for the regulation of trichome and stamen development^19–22^. In these crosstalks, the direct interaction between MYC2 and ethylene-stabilized transcription factor ETHYLENE INSENSITIVE3 (EIN3) or between JAZs and repressor of gibberellin signaling DELLA proteins are deeply involved. JA also interacts with auxin in root growth and flower development^23–25^. MYC2 also plays a role in the interaction between JA and auxin. For example, Chen *et al.* showed that apical root growth inhibition by JA is caused by the suppression of proliferative activity in root meristematic cells, and MYC2 regulates this process by repressing expression of the auxin-responsive gene *PLETHORA*, which is responsible for stem cell maintenance and cell division^25^.

Cytokinin governs plant growth and development^26^ and recent studies demonstrated that cytokinin plays a key role in the development of root vascular tissue^27–29^. In cytokinin signal transduction, hybrid histidine protein kinases perceive the cytokinin signal at the plasma membrane, and histidine phosphotransfer proteins (AHPs) transmit this signal to response regulators (ARRs) through a phosphorelay^30^. *ARRs* can be categorized into Type-B and Type-A *ARRs* encoding transcriptional activators and repressors responsible for modulating the expression of cytokinin-responsive genes. *ARR1, 2, 10, 11*, and *12* belong to Type-B and *ARR3, 4, 5, 6, 8*, and *9* belong to Type-A. In root vascular tissues, the cytokinin response occurs specifically in procambial cells. A strong cytokinin response in the procambium promotes polar auxin transport toward protoxylem precursors by controlling expression and localization of PIN-FORMED (PIN) proteins, leading to the establishment of auxin maxima in these cells^29^. The auxin response promotes xylem differentiation and suppresses the cytokinin response by inducing the expression of *ARABIDOPSIS HISTIDINE PHOSPHOTRANSFER PROTEIN 6* (*AHP6*), which encodes a pseudo-histidine phosphotransfer protein and functions as an inhibitor of cytokinin signaling. Expression of *AHP6* is essential for regulation of the cytokinin response. For example, overexpression or knock-out of A*HP6* affects the cytokinin response and plant development^31^. Several genetic studies support the essential role of the cytokinin response in vascular tissue develoment. For example, the *wooden leg* (*wol*) mutants have severe defects in cytokinin responses and display all-xylem phenotypes in their vasculature. Also, mutant plants that lack expression of Type-B *ARRs* such as *ARR1*, *ARR10*, and *ARR12* produce extra xylem^28, 29, 32^. Furthermore, treatment with exogenous cytokinin strongly suppresses the formation of xylem. These observations suggested that cytokinin is a negative regulator of xylem development^28, 29^.

Crosstalk between JA and cytokinin remains largely unknown. However, previous studies showed that environmental stresses that provoke JA responses can affect the expression of cytokinin-responsive genes^33–35^. These findings suggested that JA might interact with cytokinin to coordinate plant stress responses and growth. In this study, we showed that JA promotes xylem differentiation and a reduction of the cytokinin response underlies this process. Further molecular and genetic analysis suggested that the JA-responsive transcription factor MYC2 and the cytokinin signaling inhibitor AHP6 participate in JA-induced xylem development.

## Results

### JA interacts antagonistically with cytokinin

**Figure 1:**
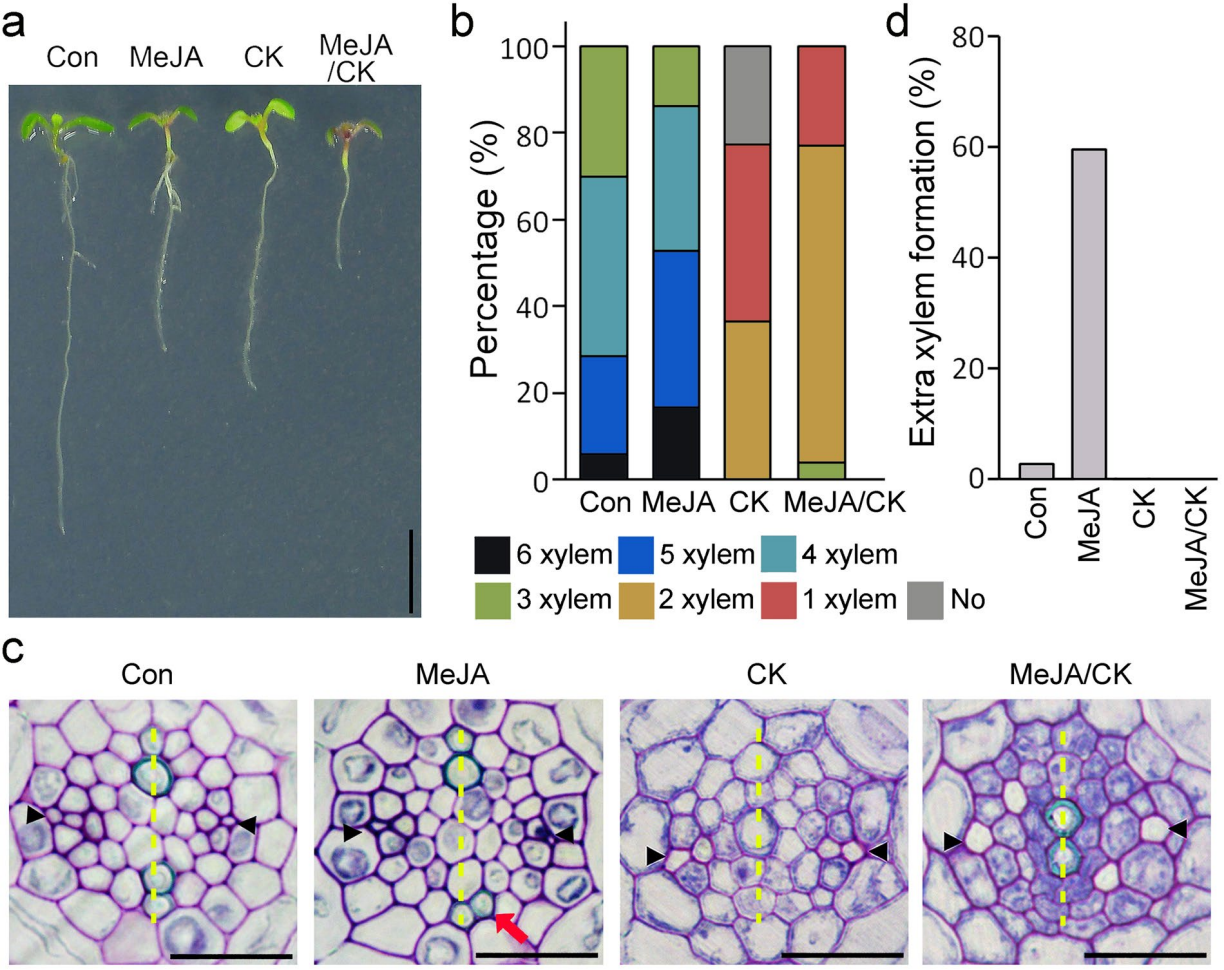
Antagonistic interaction between JA and cytokinin in xylem development. (**a**) Root growth of Col-0 grown in the indicated conditions for 7 days (MeJA, 10 μM MeJA; cytokinin, 50 nM BAP; MeJA/cytokinin, 10 μM MeJA and 50 nM BAP). (**b**) Quantification of the number of xylem cells in the xylem axis of these plants (*n* > 20). (**c**) Vasculature morphology of Col-0 grown in the indicated conditions for 7 days. Black arrows and arrowheads indicate extra xylem cells and phloem cells, respectively. The yellow dotted line indicates a xylem axis. (**d**) Quantification of extra xylem formation in these plants. Percentages were calculated by dividing the number of plants with extra xylem by the number of plants observed. Scale bar = 0.5 cm in (**a**) and 20 μm in (**c**).

Previous studies have proposed that JA and cytokinin act antagonistically in the regulation of plant development and immunity^36–38^. JA inhibits apical root growth by suppressing the activity of meristematic cells in roots^25^. To understand the regulatory interaction between JA and cytokinin, we examined root growth in Col-0 plants treated with JA, cytokinin, or both (Fig. 1a; see Supplementary Fig. S1). Treatment with JA or cytokinin alone inhibited apical growth of roots and the combined treatment inhibited root growth more severely than either treatment alone. These observations suggested that cytokinin does not nullify the negative effect of JA on apical growth of roots.

To further understand the interaction between JA and cytokinin, we analyzed the morphology of the root vasculature because cytokinin suppresses xylem formation whereas JA promotes xylem formation^39^. When we quantified the number of xylem cells in the roots of these plants by transverse sectioning and toluidine blue staining, we found that JA and cytokinin act antagonistically in xylem development (Fig. 1b). In normal growth conditions, 30%, 42%, 23%, and 5% of Col-0 roots showed 3, 4, 5, and 6 xylem cells in a xylem axis, respectively; by contrast, 15%, 33%, 36%, and 16% of JA-treated Col-0 roots formed 3, 4, 5, and 6 xylem cells, respectively, indicating that JA promotes xylem development. Unlike JA-treated or -untreated Col-0 roots, which generally had more than three xylem cells in a xylem axis, 23% of cytokinin-treated roots formed no xylem cells in the axis, 40% formed only one xylem cell, and 37% of roots formed two xylem cells, indicating that cytokinin strongly suppresses the formation of xylem. In JA/cytokinin-treated roots, all roots formed one or two xylem cells in the xylem axis, and the no-xylem phenotype was not observed, unlike Col-0 plants treated with cytokinin alone. Furthermore, we found that JA promotes formation of extra xylem adjacent to the xylem axis, which was rarely observed in JA-untreated wild-type plants (Fig. 1 c and d). The JA-induced xylem phenotype was rarely observed in the plants treated with both JA and cytokinin. These observations indicated that cytokinin diminishes the effect of JA on xylem development, suggesting an antagonistic interaction between JA and cytokinin.

To get an overview of how these hormones affect gene expression in the root, we performed RNA-sequencing (RNA-seq) using total RNA extracted from Col-0 roots treated with JA, cytokinin, or both. Results can be found in the GEO database under accession number GSE80188. When the expression patterns of 4,401 genes satisfying the criterion |fold change| ≥ 2 in at least one data set were displayed as a heat map, we found that gene expression patterns induced by cytokinin differed substantially from those induced by JA alone or by JA and cytokinin together (see Supplementary Fig. S2a). Quantitative RT-PCR analysis of cytokinin-induced gene expression partially supported this (see Supplementary Fig. S2b). JA reduced the expression of cytokinin-induced genes such as *ARRs* and *PINs*, but the roots treated with both JA and cytokinin did not show a reduction in *ARR* and *PIN* expression.

### JA promotes differentiation of xylem

**Figure 2:**
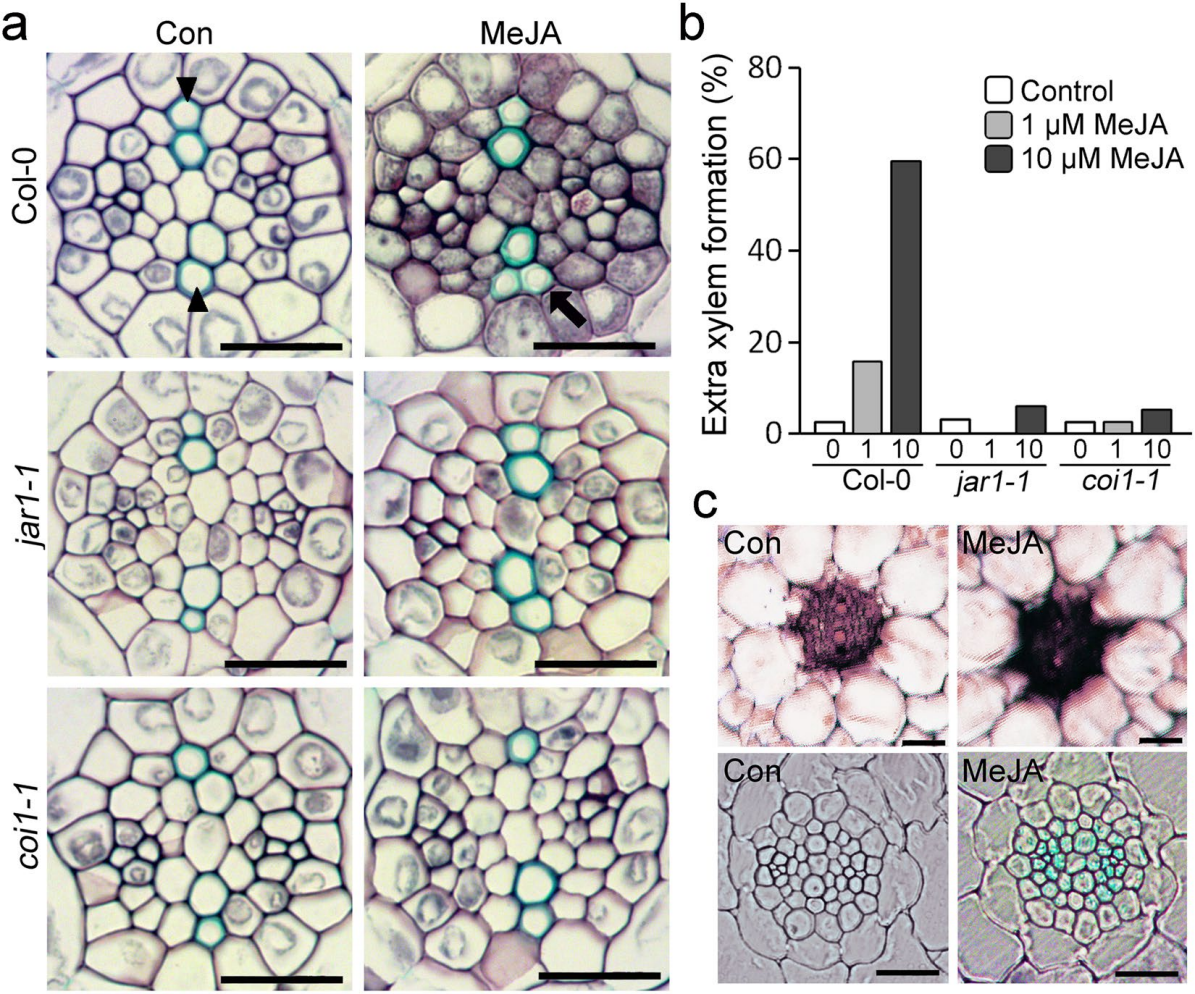
JA affects development of root vascular tissues. (**a**) Morphology of root vascular tissues of Col-0, *jar1-1*, and *coi1-1* grown in 10 μM MeJA or not treated for 7 days. Maturation regions of the indicated roots were transversely sectioned and stained with toluidine blue for detection of xylem cells. (**b**) Quantification of extra xylem formation in these plants (*n* > 30). Percentages were calculated by dividing the number of plants with extra xylem by the number of plants observed. (**c**) RNA *in situ* hybridization and GUS staining showing JA responses in vascular tissues. Spatial expression pattern of the JA-responsive gene *VSP1* in roots (top) grown in 10 μM MeJA (right) or not treated (left) for 7 days. GUS staining analysis (bottom) was performed using *4XJARE::GUS* plants in which *GUS* expression was controlled by the activity of 4 copies of the Jasmonic Acid Response Element (JARE). These plants were treated with 100 μM MeJA for 6 hrs. GUS staining solution with 1 mM ferrocyanide/ferricyanide was used. Arrow and arrowheads indicate extra xylem and protoxylem, respectively. Scale bar = 20 μm.

To further understand the effect of JA on the development of extra xylem, we examined the development of root vascular tissue in wild type and the JA signaling-defective mutants *jar1-1* and *coi1-1* (Fig. 2a and b). Transverse sectioning and toluidine blue staining showed that the morphology of root vascular tissue was almost identical between wild type and JA-signaling defective mutants grown in the absence of exogenous JA. However, around 15% of wild-type plants treated with 1 μM JA (Col-0, *n* = 32) developed extra xylem neighboring the protoxylem of a xylem axis. Around 60% of Col-0 plants (*n* = 37) developed extra xylem when grown in 10 μM JA. However, unlike Col-0 plants, in the *jar1-1* and *coi1-1* mutants, JA did not affect the morphology of root vascular tissues, and the extra-xylem phenotype was rarely detected in these mutant plants. However, the JA-biosynthesis mutant *opr3* did form extra xylem in response to JA (see Supplementary Fig. S3). These observations suggested that JA signaling affects vascular tissue morphology in response to JA.

Examination of the JA response domain in the roots supported this (Fig. 2c). RNA *in situ* hybridization of the JA-induced gene *VEGETATIVE STORAGE PROTEIN1* (*VSP1*) in Col-0 roots showed that vascular tissues had a stronger signal for the *VSP1* transcripts, compared with other tissues. JA treatment increased accumulation of the *VSP1* transcript in the vascular tissues. Additionally, transgenic plants expressing a *GUS* reporter gene under the control of the Jasmonic Acid Response Element (*4XJARE::GUS*) displayed GUS staining mainly in the vascular tissues in response to JA.

To characterize the effect of JA on xylem development, we counted the number of vascular tissue cells, and found that there was no significant difference in the number of vascular tissue cells between Col-0 roots grown with and without JA (see Supplementary Fig. S4a). However, the number of xylem cells in Col-0 roots grown in 10 μM JA was significantly higher than that of Col-0 grown without JA (see Supplementary Fig. S4b). These observations suggested that the JA response promotes xylem differentiation.

### JA reduces the cytokinin response in root vascular tissue

**Figure 3:**
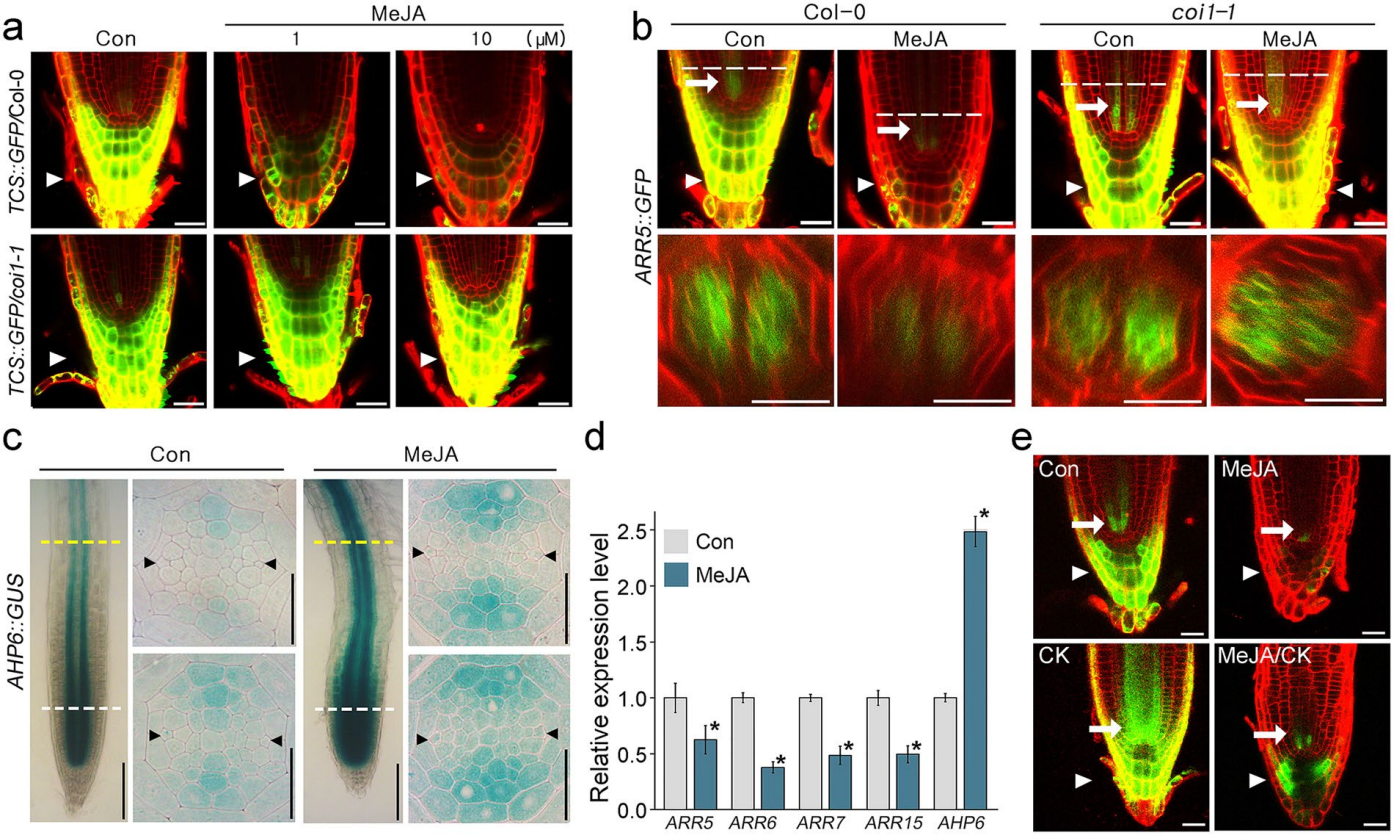
JA reduces the cytokinin response. (**a**) Effect of JA on the cytokinin response was analyzed by visualizing the fluorescent signals in *TCS::GFP/*Col-0 (top) and *TCS::GFP/coi1-1* (bottom) plants grown in 1 and 10 μM MeJA for 7 days. (**b**) Confocal longitudinal (top) and cross (bottom) section images showing that JA suppressed cytokinin-induced *ARR5* expression in root vascular tissues of Col-0, but not in *coi1-1* mutants. The indicated plants were grown in 10 μM MeJA or not treated for 7 days. (**c**) GUS staining of *AHP6::GUS* plants grown in 10 μM MeJA or not treated for 7 days using GUS staining solution without ferrocyanide/ferricyanide. (**d**) Expression levels of cytokinin-responsive genes measured by qRT-PCR. Total RNA was extracted from Col-0 roots grown in the indicated conditions for 7 days. *GAPDH* was used as a reference gene for normalization. Error bars represent S.D. and asterisks indicate statistically significant differences between the corresponding samples and their control (*p* < 0.01, *t*-test). (**e**) Visualization of the fluorescent signals in *ARR5::GFP/*Col-0 grown in the indicated conditions (MeJA, 10 μM MeJA; cytokinin, 50 nM BAP; MeJA/cytokinin, 10 μM MeJA and 50 nM BAP). White arrows and arrowheads indicate cytokinin response in the vascular tissue and root cap, respectively. Dotted lines indicate the longitudinal position where confocal optical cross-sectioning was performed. Scale bar = 100 μm in whole-mounted images of (**c**) and 20 μm in others.

Cytokinin negatively regulates xylem development^28, 29^. Because JA promotes xylem development, we hypothesized that JA might affect cytokinin responses in the root vasculature. To address this, we tested whether JA causes changes in the cytokinin response in transgenic plants containing the *TCS::GFP* reporter for the cytokinin response^2^ (Fig. 3a). In JA-untreated conditions, we detected strong fluorescent signals in *TCS::GFP* root caps, but JA treatment decreased the intensity of the fluorescent signal in a dosage-dependent manner. Unlike wild-type plants, *coi1-1* mutants did not respond to JA, and the suppression of GFP signals by JA was not observed in the mutant plants. These observations suggested that the JA response suppresses the cytokinin response. However, changes in the cytokinin response in root vasculature were not obvious in this system because the fluorescent signals in the tissues were too weak to visualize.

We also tested the *ARABIDOPSIS RESPONSE REGULATORS5* (*ARR5*) and *WOODEN LEG* (*WOL*) promoters, two other cytokinin-responsive markers^28, 40^. In *ARR5::GFP* transgenic plants not treated with JA, fluorescent signals were observed in root caps and vascular tissues. In *ARR5::GFP* plants grown with JA, the GFP signals were much weaker than in *ARR5::GFP* plants grown without JA (Fig. 3b). Furthermore, optical sectioning showed that JA treatment decreased the intensity of fluorescent signals in vascular tissues of *ARR5::GFP* plants. However, in the *coi1-1* background, JA did not affect the fluorescent signals. Similar to *ARR5::GFP* transgenic plants, *WOL::GFP/Col-0* plants displayed suppressed fluorescent signals in response to JA but *WOL::GFP/coi1-1* plants did not (see Supplementary Fig. S5). These results suggested that the JA response suppresses the cytokinin response in root vascular tissues. *AHP6* inhibits the cytokinin response and *AHP6* expression is negatively correlated with cytokinin responses^28, 29^. In contrast to *ARR5* and *WOL*, expression of *AHP6* increased in response to JA. In JA-untreated *AHP6::GUS* seedlings, GUS staining was predominantly detected in the protoxylem and neighboring pericycle cells. However, JA treatment expanded the *AHP6* expression domain to the procambial cells (Fig. 3c; see supplementary Figs S6 and S7). These results suggested that JA suppresses the cytokinin response in the root vasculature. Quantitative RT-PCR analysis supported this idea (Fig. 3d). Expression levels of Type-A *ARRs* such as *ARR5*, *6*, *7*, and *15* were lower in Col-0 roots grown with JA than those grown without JA, whereas the expression level of *AHP6* was higher in the JA-treated roots. Unlike Type-A *ARRs*, expression of Type-B *ARRs* in JA-treated Col-0 plants tended to be slightly upregulated compared to those in JA-untreated Col-0 (see Supplementary Fig. S8). Previous studies showed that expression levels of Type-A *ARRs* increased in response to cytokinin, but expression of Type-B *ARRs* tended to decrease^41^. These results suggested that the decreased expression of Type-A *ARRs* and the increased expression of Type-B *ARRs* might be caused by suppression of the cytokinin response by JA. When the fluorescent signals were visualized in *ARR5::GFP* plants treated with JA, cytokinin, or both, the intensity of fluorescent signal in JA/cytokinin-treated *ARR5::GFP* plants was higher than that in the JA-treated *ARR5::GFP* plants, but lower than that in cytokinin-treated *ARR5::GFP* plants. These observations supported the negative effect of JA on cytokinin responses and the antagonistic relationship between JA and cytokinin (Fig. 3e).

**Figure 4:**
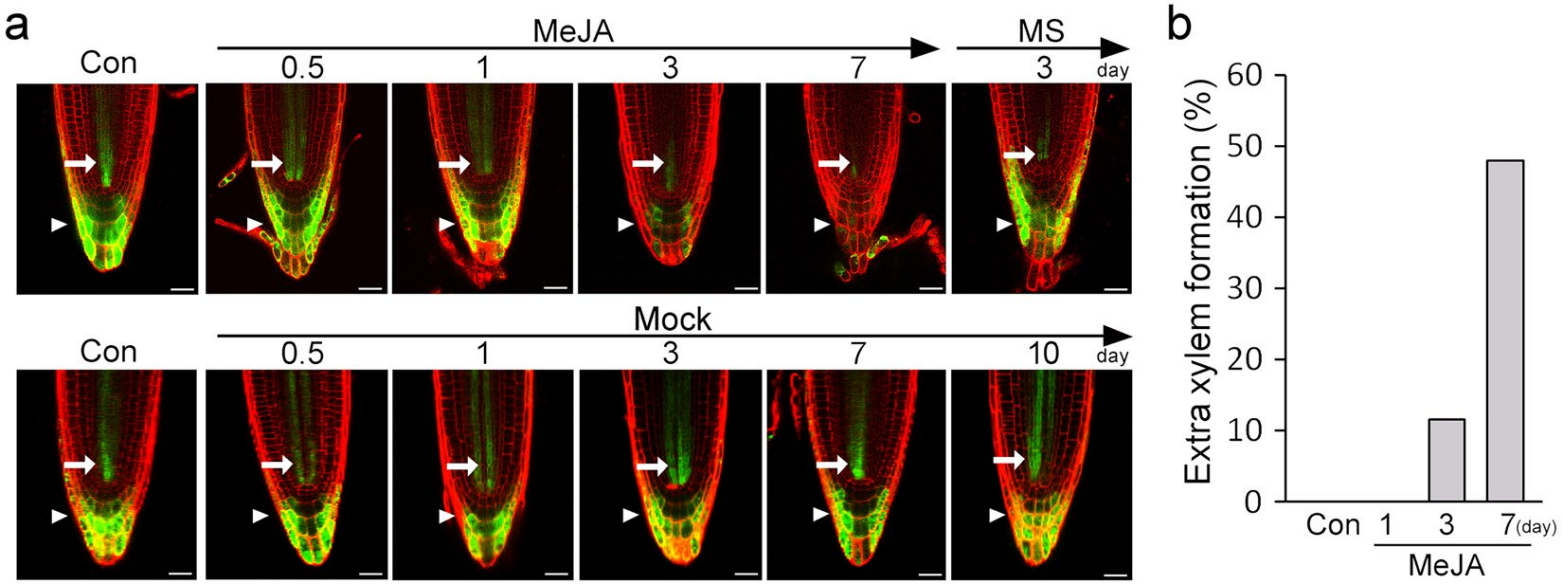
Gradual suppression of the cytokinin response by JA. (**a**) Suppression of the cytokinin response by JA was analyzed in a time course by monitoring fluorescent signals in *ARR5::GFP* plants. Five-day-old *ARR5::GFP* plants grown without JA were transferred to MS media containing 10 μM MeJA, and grown for the indicated times. Mock indicates MS media containing 0.05% EtOH. White arrows and arrowheads indicate cytokinin response in the vascular tissue and root cap, respectively. (**b**) Quantification of extra xylem formation in these plants (*n* > 20). Percentages were calculated by dividing the number of plants with extra xylem by the number of plants observed. Scale bar = 20 μm.

To further understand the reduction of the cytokinin response by JA, we analyzed changes in the cytokinin response in a time course in the *ARR5::GFP* roots treated with JA (Fig. 4a). The fluorescent signals in the roots exposed to JA for 0.5 or 1 day were almost identical to those in JA-untreated roots. However, as the exposure time increased, the intensity of the fluorescent signals gradually decreased. In the roots exposed to JA for 3 days, the fluorescent signals were obviously weaker than those in control roots and the signals almost disappeared at 7 days. When these roots were transferred to media without JA and grown for 3 days, the fluorescent signals reappeared. To understand the relationship between cytokinin responses and extra xylem formation, we quantified formation of extra xylem in these plants (Fig. 4b). The extra-xylem phenotype was rarely detected in the roots exposed to JA for 1 day, but the roots exposed to JA for 3 days or 7 days showed the extra-xylem phenotype. These observations suggested that suppression of the cytokinin response by JA does not occur rapidly, but the JA-mediated xylem phenotype is caused by suppression of the cytokinin response.

### Overexpression of *AHP6* promotes formation of extra xylem

**Figure 5:**
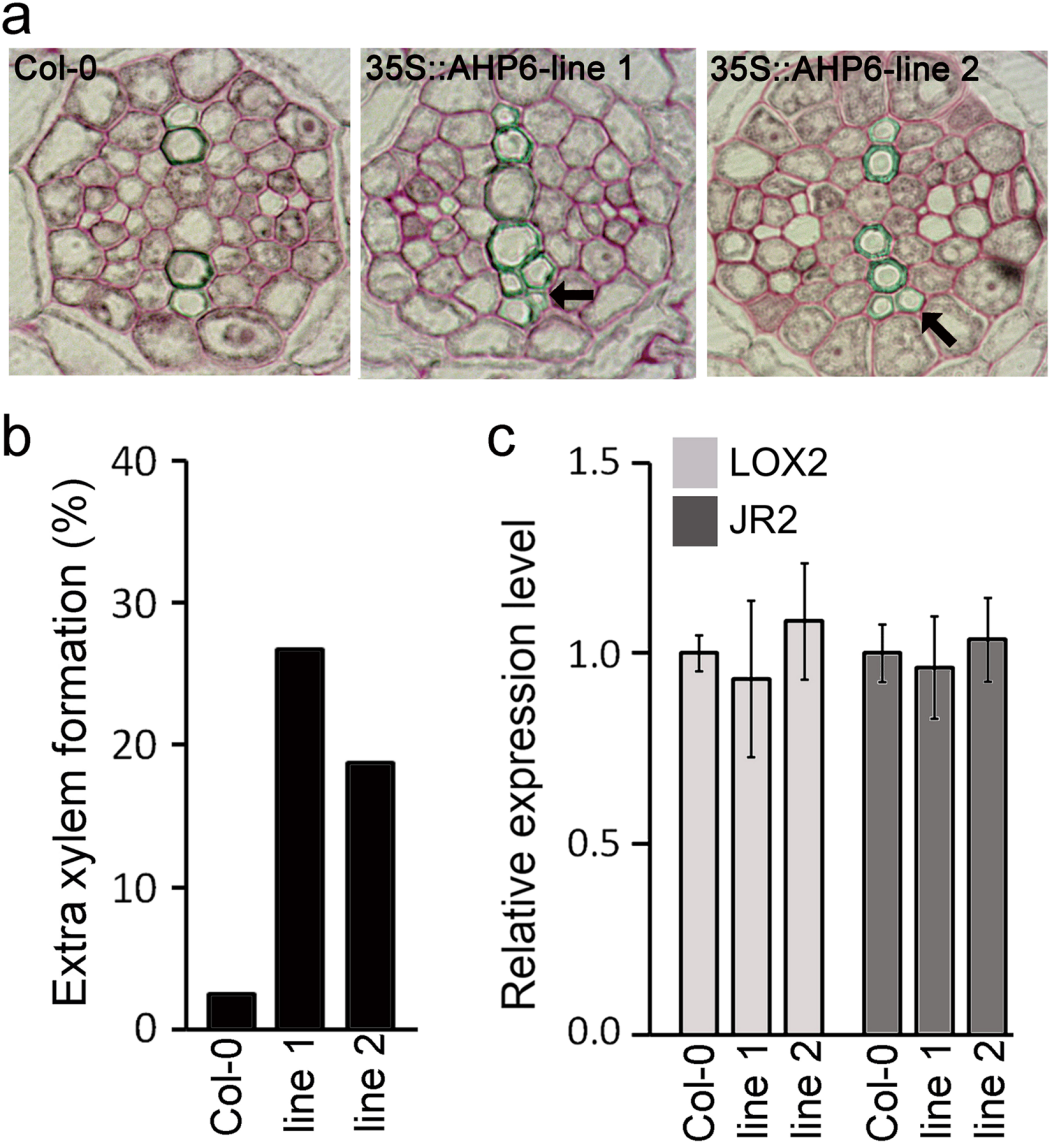
Reduction of the cytokinin response promotes the formation of extra xylem. (**a**) Cross section images of *35S::AHP6* roots grown in MS media for 7 days. (**b**) Quantification of extra xylem formation in these plants (*n* > 30). Percentages were calculated by dividing the number of plants with extra xylem by the number of plants observed. (**c**) Expression levels of the JA-induced genes *LOX2* and *JR2* in these plants. Line 1 and 2 indicate individual lines of *35S::AHP6* transgenic plants. Error bars represent S.D. *GAPDH* was used as a reference gene. Black arrows indicate extra xylem cells. Scale bar = 20 μm.

To understand whether the low cytokinin response can promote the development of extra xylem, we analyzed xylem development in *AHP6*-overexpressing transgenic plants, which have reduced cytokinin responses (see Supplementary Fig. S9). Further characterization showed that around 20–27% of *35S::AHP6* plants form extra xylem, even in the absence of exogenous JA, suggesting that a reduction of cytokinin can induce the formation of extra xylem (Fig. 5a and b). The *35S::AHP6* and wild-type plants also showed similar numbers of vascular cells; however, the *35S::AHP6* plants tended to have more xylem cells, compared to wild-type plants (see Supplementary Fig. S10). We then checked the JA response in *AHP6*-overexpressing transgenic plants by analyzing expression levels of the JA-induced genes *LIPOXYGENASE2* (*LOX2*) and *JASMONATE-RESPONSIVE2* (*JR2*). We found no difference in the expression levels of *LOX2* and *JR2* between wild-type and *AHP6*-overexpressing plants (Fig. 5c). These findings indicated that a low cytokinin response promotes xylem development without affecting JA responses.

**Figure 6:**
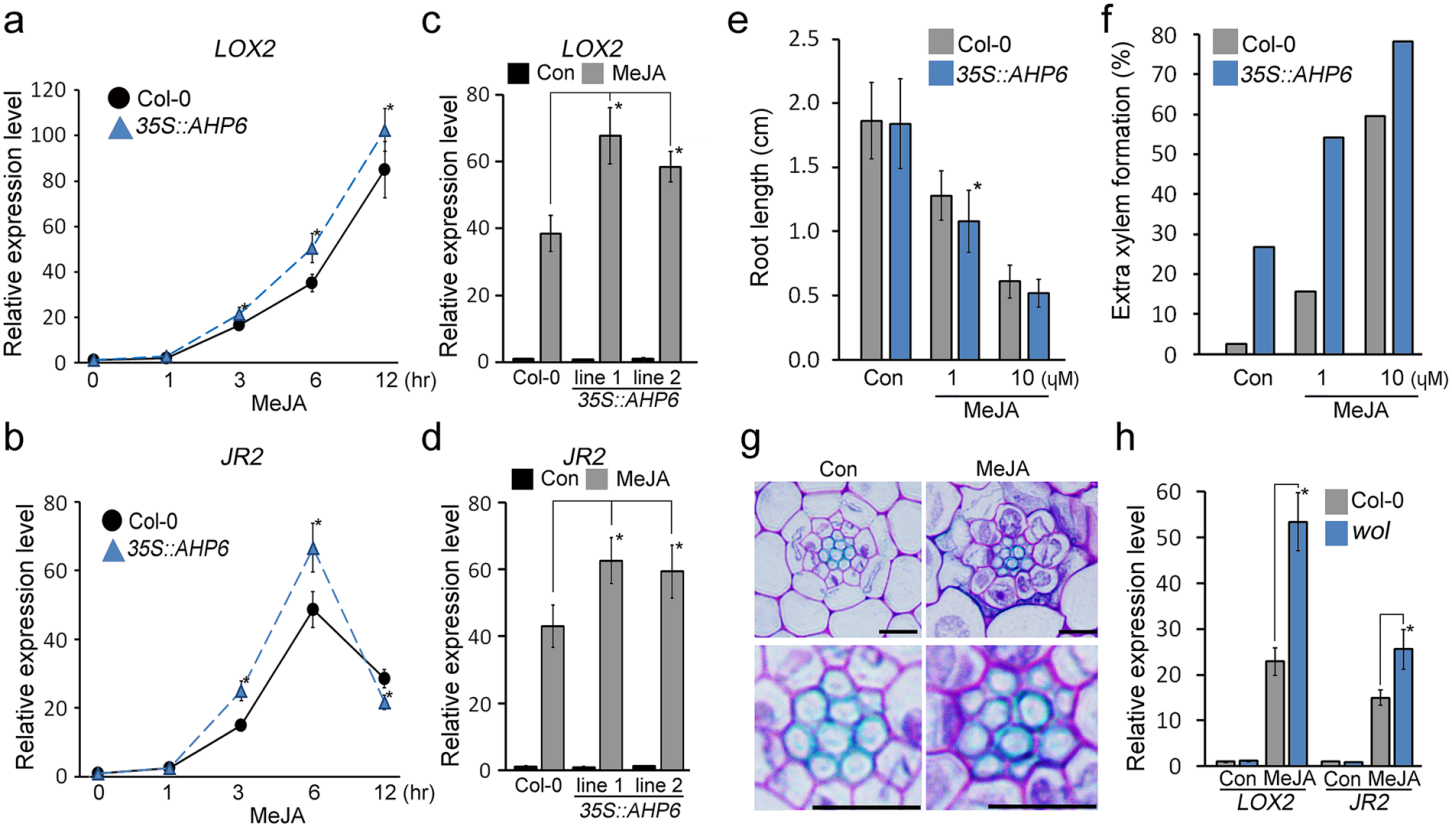
JA responses in plants with reduced cytokinin responses. *LOX2* (**a**) and *JR2* (**b**) expression was analyzed in *35S::AHP6* plants by qRT-PCR. Seven-day-old *35S::AHP6* and Col-0 seedlings were transferred to media containing 100 μM MeJA and incubated for the indicated time. Total RNA was extracted from these roots. Expression levels of *LOX2* (**c**) and *JR2* (**d**) in two independent lines of 7-day-old *35S::AHP6* plants treated with 100 μM JA for 6 hrs. (**e**) Root growth of *35S::AHP6* plants grown in JA-treated and -untreated conditions for 7 days (*n* > 30). (**f**) Quantification of extra xylem formation in these plants (*n* > 29). Percentages were calculated by dividing the number of plants with extra xylem by the number of plants observed. (**g**) Root vasculature (top) and high-resolution images (bottom) of *wol* mutants grown in 10 μM MeJA-untreated (left) or -treated (right) conditions for 12 days. (**h**) Expression levels of *LOX2* and *JR2* in *wol* mutant roots. Ten-day-old *wol* and Col-0 seedlings were transferred to MS media containing 100 μM MeJA and incubated for 3 hrs. Total RNA was extracted from the roots of these plants. *GAPDH* was used as a reference gene. Error bars represent S.D. and asterisks indicate statistically significant differences between the corresponding samples and their control (*p* < 0.01, *t*-test). Scale bar = 10 μm.

To understand the regulatory interaction between JA and cytokinin, we checked JA-induced changes in the expression of JA-induced genes in *35S::AHP6* plants in a time course (Fig. 6a–d). In JA-untreated conditions, *LOX2* and *JR2* expression was almost identical between wild-type and *AHP6*-overexpressing plants. However, *LOX2* and *JR2* expression in JA-treated *35S::AHP6* plants was higher than that in JA-treated Col-0 plants, and the difference tended to increase with increasing treatment time. These results suggested that *35S::AHP6* plants show higher JA responses when treated with JA. To explore this, we analyzed root growth inhibition and extra xylem formation in JA-treated *35S::AHP6*. In JA-untreated conditions, the root length of *35S::AHP6* plants was similar to that of wild type. However in JA-treated conditions, the root length of *35S::AHP6* plants was shorter than that of wild-type plants (Fig. 6e). When extra xylem formation was quantified, approximately 54% and 78% of *35S::AHP6* plants showed extra xylem in response to 1 µM and 10 µM JA, respectively, while around 15% and 60% of wild-type plants did (Fig. 6f). These results suggested that the *35S::AHP6* plants might be more sensitive to JA.

The *wol* mutants have severely compromised cytokinin responses and defects in root growth^27, 29^. The *wol* mutants have fewer vascular cells compared with wild-type plants, and all root vascular cells differentiate into xylem cells^27, 29^. As expected, the root vascular morphology of JA-treated *wol* mutants was almost identical to JA-untreated *wol* mutants, and the number of vascular tissue cells all of which were xylem cells was similar between them (Fig. 6g; see Supplementary Fig. S11). The expression of *LOX2* and *JR2* was almost identical between JA-untreated wild type and *wol* mutants. However, in JA-treated conditions, expression levels of *LOX2* and *JR2* were higher in in *wol* mutants than in wild-type, suggesting that *wol* mutants show higher JA responses when treated with JA (Fig. 6h).

We also checked cytokinin responses in the JA-signaling mutant *jar1-1* by analyzing the expression level of *ARR5* (see Supplementary Fig. S12). In cytokinin-untreated or -treated conditions, the expression levels of *ARR5* were similar between wild-type and *jar1-1* mutant plants grown in the same conditions. To verify this, we quantified the expression level of *ARR5* in another JA-signaling mutant, *myc2-3*. Similar to *jar1-1*, the *myc2-3* mutants exhibited similar cytokinin responses to wild-type plants in cytokinin-untreated or -treated conditions. These observations suggested that JA signaling mutants and wild-type plants have similar sensitivities to cytokinin.

### Expression of *AHP6* and *MYC2* is involved in the JA-mediated xylem phenotype

**Figure 7:**
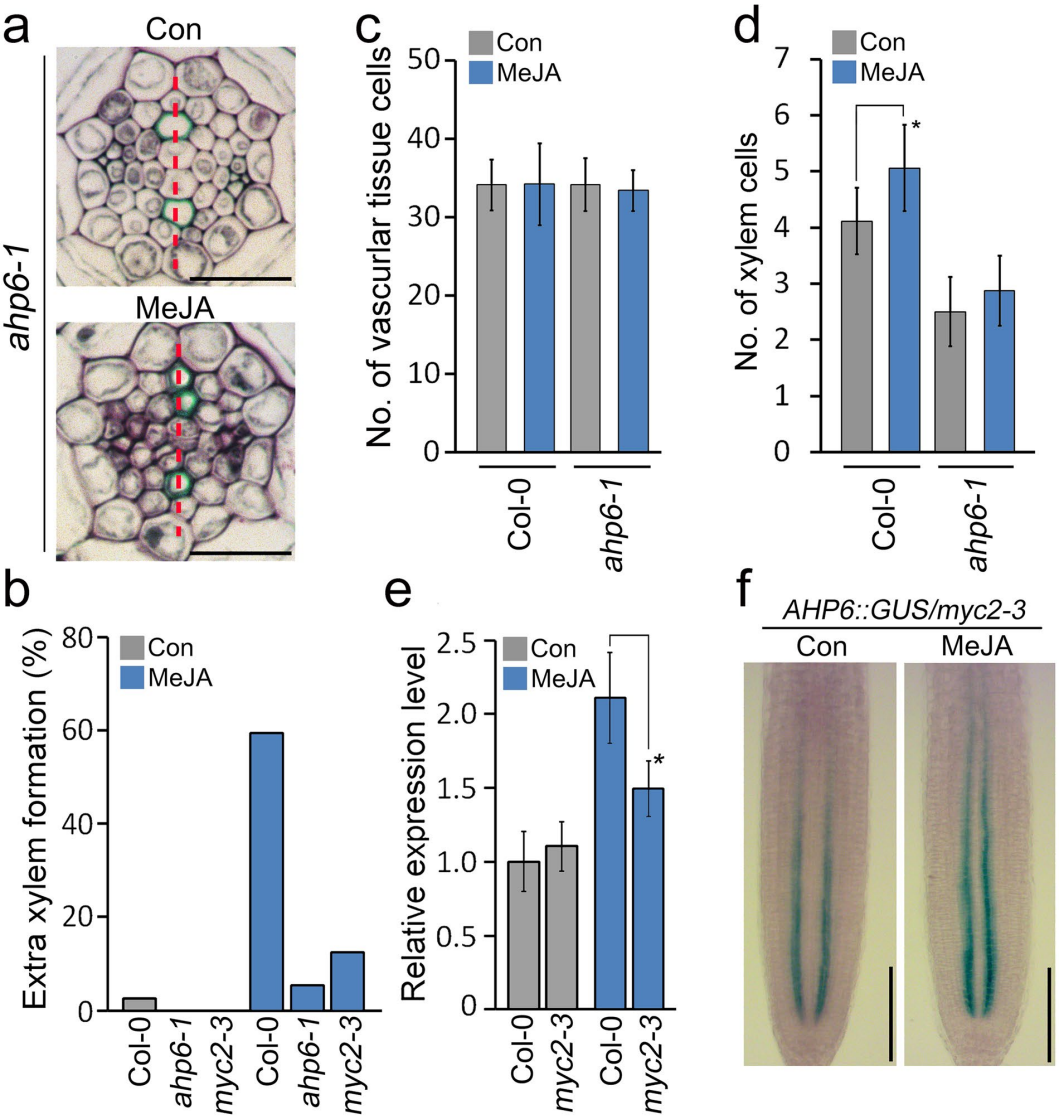
*AHP6* and *MYC2* are involved in JA-mediated xylem development. (**a**) Root vasculature of *ahp6-1* plants grown in 10 μM MeJA-untreated (top) or -treated (bottom) conditions for 7 days. The red dotted line indicates a xylem axis. (**b**) Quantification of extra xylem formation in the roots of Col-0, *ahp6-1*, and *myc2-3* mutants grown in 10 μM MeJA or not treated for 7 days (*n* > 22). (**c** and **d**) The number of vascular tissues cells (**c**) and xylem cells (**d**) in JA-treated and -untreated *ahp6-1* mutant plants. (**e**) Analysis of *AHP6* expression in *myc2-3* mutants by qRT-PCR. Total RNA was extracted from Col-0 and *myc2-3* roots grown in 10 μM MeJA or not treated for 7 days. *GAPDH* was used as a reference gene. Error bars represent S.D. and asterisks indicate statistically significant differences between the corresponding samples and their control (*p* < 0.01, *t-*test). (**f**) GUS staining of *AHP6::GUS/myc2-3* plants grown in 10 μM MeJA or not treated for 7 days using 1 mM ferrocyanide/ferricyanide-containing GUS staining solution. Scale bar = 20 μm in (**a**) and 100 μm (**d**).

Since JA expanded the *AHP6* expression domain to the procambium, where extra xylem formed, we explored the possible involvement of *AHP6* in JA-mediated xylem development. To do this, we analyzed xylem development in *ahp6-1* mutants grown with and without JA. Consistent with a previous study by Mähönen *et al.*, development of protoxylem was suppressed in *ahp6-1* mutants grown without JA^28^ (Fig. 7a and b). Furthermore, unlike wild-type plants that formed extra xylem cells in response to JA, *ahp6-1* mutants did not produce, or rarely produced extra xylem cells adjacent to a xylem axis in response to JA. Counting the number of vascular tissue cells and xylem cells in *ahp6-1* mutants showed no significant difference between JA-untreated and JA-treated *ahp6-1* mutants. These findings suggested that *AHP6* is deeply involved in the JA-induced extra-xylem phenotype (Fig. 7c and d).

We also found that *MYC2* is involved in JA-mediated xylem development. In JA-untreated conditions, *MYC2*-overexpressing transgenic plants did not show an extra-xylem phenotype, similar to Col-0. However in 1 µM JA-treated conditions, approximately 50% of *35S::MYC2* transgenic plants exhibited an extra-xylem phenotype and the rate was around 3-fold higher than in wild-type plants grown in the same conditions. This suggested that overexpression of *MYC2* promotes the formation of extra xylem in response to JA, and *MYC2* is involved in JA-mediated modulation of xylem development (see Supplementary Fig. S13). To explore this, we examined xylem development in *myc2-3* mutants and found that formation of extra xylem is inhibited in *myc2-3* mutants (Fig. 7b), indicating that *MYC2* mediates xylem development in response to JA.

When we compared *AHP6* expression between Col-0 and *myc2-3* mutants grown without JA, we observed no significant difference in *AHP6* expression. However, in JA-treated conditions, the expression of *AHP6* in *myc2-3* mutants was lower than that in wild type (Fig. 7e). These results suggested that MYC2 is involved in the regulation of *AHP6* expression in response to JA. GUS staining of *AHP6::GUS/myc2-3* plants supported this, as *myc2-3* plants grown with and without JA showed similar GUS staining (Fig. 7f). Promoter analysis predicted that the *AHP6* promoter contains a putative MYC2-binding sequence, (CACATG, at -1623 bp) (see Supplementary Table S1). A yeast one-hybrid assay suggested that MYC2 possibly binds to the *AHP6* promoter region containing the MYC2-binding sequence (see Supplementary Fig. S14). However, ChIP analysis using *35S::MYC2-GFP* transgenic plants showed that MYC2 does not directly interact with the *AHP6* promoter.

## Discussion

Developmental flexibility under stress conditions largely occurs via the interaction between hormones that mediate stress responses and developmental processes. JA coordinates the dynamics of plant stress responses and growth through interactions with other phytohormones such as gibberellic acid and auxin^21, 25, 42^. Growing numbers of studies have proposed that JA also interacts with cytokinin in the regulation of plant development and physiology. For example, JA prevents cytokinin-induced soybean callus growth^43^ and also inhibits the effect of cytokinin on chlorophyll degradation and the plant immune system^36, 37^. Expression of genes involved in these processes is regulated differently by JA and cytokinin. Furthermore, each treatment nullifies the effect of the other. A recent study of Nitschke *et al.* also supports the interaction of JA and cytokinin in circadian stress responses^38^. In this study, we showed that JA signaling promotes xylem differentiation in response to JA, and the antagonistic interaction with cytokinin is involved in this process. Cytokinin is a key negative regulator of xylem development^27–29^. For example, cytokinin signaling-defective *wol* plants develop small numbers of procambial cell files due to suppression of procambial cell division^27^. Moreover, this mutant produces all protoxylem in its root vascular tissues^29^. In this study, we showed that JA induces formation of extra xylem in wild-type plants, but not in JA-signaling mutants such as *coi1-1* and *jar1-1*. These suggest that JA response promotes xylem development in response to JA.

Analysis of the cytokinin response showed that the reduction of the cytokinin response is responsible for the JA-mediated xylem phenotype. Expression of cytokinin-induced genes was downregulated by JA, and cytokinin treatment diminished the effect on JA on formation of extra xylem and expression of cytokinin-induced genes. Additionally, *35S::AHP6* plants with reduced cytokinin responses formed extra xylem even in the absence of JA. When expression levels of JA-responsive genes were analyzed in the *35S::AHP6* and wild-type grown in JA-untreated conditions, we found no difference between them. However *35S::AHP6* plants exhibited increased expression of these genes compared to wild type in JA-treated conditions, suggesting that *35S::AHP6* plants with reduced cytokinin responses show higher JA responses than wild-type plants when treated with JA. Furthermore, root growth inhibition and extra xylem development is promoted in these transgenic plants compared to wild-type plants. Previous studies reported that plants with higher JA responses show enhanced tolerance to environmental stresses such as drought and salt^44–48^. Additionally, a study by Nishiyama *et al.* showed that cytokinin-deficient transgenic plants are also resistant to these environmental stresses^49^. Collectively, these suggest that JA antagonistically interacts with cytokinin and the plants with reduced cytokinin responses are more sensitive to JA.

In this study we also showed that the expression level of *AHP6* in two independent *35S::AHP6* lines, was approximated 36- and 28-fold higher than in wild-type plants, respectively. Extra xylem was observed in around 22% and 17% of the transgenic plants, respectively, suggesting a positive relationship between *AHP6* expression and extra xylem formation. Together with the results that *ahp6-1* rarely formed extra xylem in response to JA, these observations suggest that *AHP6* is involved in the JA-mediated xylem phenotype. When the expression level of *AHP6* was compared between JA-untreated *35S::AHP6* and JA-treated Col-0 plants, the *35S::AHP6* plants showed about 15-fold higher expression of *AHP6* than JA-treated Col-0. However, the extra-xylem phenotype was observed in around 20% of *35S::AHP6* and 60% of wild-type plants, indicating that *AHP6* expression is not tightly linked to the JA-mediated formation of extra xylem although expression of *AHP6* is essential for the formation of extra xylem in response to JA. These findings suggest that other JA-regulated factors are involved in this process together with *AHP6*.

MYC2 plays a key role in the regulation of JA responses^11, 50^. Chen *et al.* reported that MYC2 mediates the inhibition of apical root growth in response to JA by suppressing expression of the auxin-responsive gene *PLETHORA* ^25^, suggesting that MYC2 plays an essential role in modulation of root development in response to JA. In this study, we suggest that MYC2 is also responsible for JA-mediated xylem development. Indeed, formation of extra xylem was strongly suppressed in *myc2-3* mutants. These findings suggest that the MYC2 transcription factor mediates xylem differentiation as well as apical growth of roots in response to JA. When expression of endogenous *AHP6* was analyzed in *myc2-3* and wild-type plants exposed to JA, the *myc2-3* mutants exhibited reduced expression of *AHP6* compared to wild-type plants. These findings suggest that the MYC2 transcription factor promotes *AHP6* expression in response to JA. However, based on our ChIP results, it is not likely that MYC2 directly regulates expression of *AHP6* and downstream regulators of MYC2 might be involved in the direct regulation of *AHP6* expression.

Plants dynamically coordinate their growth and defenses in response to changes in environmental conditions, and it has been thought that growth inhibition is one of the adaptations that help plants survive environmental stresses^51^. In this study we showed that JA promotes differentiation of meristematic procambial cells to xylem cells. When we analyzed root development in wild-type plants grown under drought stress, drought inhibited root growth and promoted the formation of extra xylem, as JA does (see Supplementary Fig. S15). This suggests that promotion of xylem differentiation from meristematic cells might be one of the developmental mechanisms that plants use to inhibit their growth and thus to survive environmental stresses. Previous studies showed that JA or JA-dependent environment stresses can affect the expression of genes involved in cytokinin responses^33–35^ partially support this hypothesis. Collectively, our study proposes that the interaction of JA and cytokinin is involved in coordinating the dynamics of plant growth and defense under environmental stresses. Further molecular and genetic approaches will expand our understanding of the mechanisms of the regulatory interaction between JA and cytokinin.

## Materials and Methods

### Plant materials, growth and treatment

Plants of the *Arabidopsis thaliana* ecotype Columbia (Col-0) were used as controls in this study. The *jar1-1*, *coi1-1, myc2-3, wol*, *ahp6-1*, *4XJARE::GUS, ARR5::GFP, TCS::GFP*, *AHP6::GFP* and *35S::MYC2-GFP* plants have been described previously^2, 14, 27–29, 40, 52–54^. These seeds were obtained from the Nottingham Arabidopsis Stock Centre (NASC) or kind donation from Dr. Helariutta, Dr. Mähönen, and Dr. Chua. Seeds were surface sterilized, and plated on ½-strength Murashige and Skoog (1/2x MS) solid media. After 2 days of vernalization at 4 °C in darkness, plants were grown vertically with a light regime of 16/8 hours (light/dark) at 22 °C. For drought treatment, PEG-containing media was prepared as previously described^55^.

### Plasmid construction

For overexpression of *AHP6*, the *35S::AHP6* construct was generated using the GATEWAY system (Invitrogen). The *AHP6* cDNA fragment was amplified by PCR using total RNA extracted from 7-day-old Arabidopsis roots. The pENTRY-AHP6 plasmid was generated by inserting the amplified cDNA fragments into the pDONR221 vector (Invitrogen) using the BP reaction. The pENTRY-AHP6 construct was then recombined into the modified pMDC plant binary vector carrying the 35S promoter through the LR reaction. For the *AHP6::GUS* construct, the *AHP6* promoter (1877 bp) was isolated by PCR from Arabidopsis genomic DNA. This DNA fragment was inserted into the *Pst*I/*Eco*RI-digested pCAMBIA vector containing *β-glucuronidase* (*GUS*) by the Gibson Assembly Cloning system (New England BioLabs). Both constructs were introduced into Arabidopsis Col-0 plants by the floral dip method.

### Embedding, sectioning, and staining

Technovit embedding and sectioning were performed as described^56^ with slight modifications. Arabidopsis roots were fixed in 4% paraformaldehyde for 2 h and then washed in ddH_2_O three times for 1 h each. The fixed samples were dehydrated in an ethanol series (20, 40, 60, 80, and 100% (v/v) in ddH_2_O). The dehydrated samples were sequentially incubated in a series of Technovit 7100 cold-polymerizing resin (33, 66, and 100% (v/v) in EtOH) for 3 h each. Samples were further incubated in 100% Technovit resin for 1 day and solidified with a 15:1 (v/v) mixture of Technovit and hardener solution II at room temperature in a mold for 1 day. Sections (3–4 μm) were taken from the maturation zone of roots (around 2.5–3 mm above the root tip for JA-untreated plants, and around 2–2.5mm above the root tip for JA-treated plants with shorten roots). Dehydrated sections were stained with 0.05% toluidine blue solution to detect xylem cells (pH 4.5).

### GUS staining

GUS staining was performed as described^57^ with slight modifications. *AHP6::GUS/Col-0* and *AHP6::GUS/myc2-3* grown in JA-treated or -untreated conditions for 7 days were incubated in GUS staining solution with or without 1 mM ferrocyanide/ferricyanide (100 mM NaPO_4_ pH 7.0, 0.5 mM 5-bromo-4-chloro-3-indolyl-glucuronide, and 0.2% Triton X-100) at 37 °C for 3 or 8 h. The samples were then washed with 100 mM NaPO_4_ (pH 7.0) and incubated in 70% ethanol at 4 °C overnight.

### Quantitative RT-PCR

Quantitative RT-PCR analyses were performed using total RNA extracted from roots. Total RNA extraction was carried out using the RNeasy plant mini-prep kit (Qiagen) according to the manufacturer’s instructions. For the first-strand cDNA synthesis, 20 µL of reverse transcription reaction was performed using 2 µL of total RNA and Superscript III reverse transcriptase (Invitrogen). For quantitative PCR, a master mix was prepared using a LightCycler 480 SYBR GREEN I Master (Roche). PCR reactions and fluorescence detection were performed using a LightCycler NANO Real-Time PCR machine (Roche). PCR conditions were programmed according to the manufacturer’s instructions (initial denaturation at 95 °C for 5 min, denaturation at 95 °C for 10 sec, annealing at 58 °C for 10 sec, and extension at 72 °C for 10 sec with 45 cycles). Expression levels were analyzed using at least two biological and three technical replicates. *AtGAPDH* (*At1G13440*) was used as an internal control. Primer sequence information is available in Supplementary Table S2.

### RNA *in situ* hybridization

RNA *in situ* hybridization was carried out as described by Takechi *et al.* with slight modification^58^. The sections (4–6 mm thick) were collected using a rotary microtome and these sections were stretched on the surface of a glass slide using DEPC-treated water. *VSP1*-specific sense and antisense probes were labeled using the DIG RNA Labeling Kit (Roche) according to the manufacturer’s protocol. Ten ng of DIG-labeled probe was used for the hybridization.

### Yeast one-hybrid assay

To investigate the direct interaction between MYC2 and the *AHP6* promoter, a yeast one-hybrid assay (Clontech) was performed according to the manufacturer’s instructions. The *AHP6* promoter and full-length *MYC2* cDNA were amplified by PCR and inserted into the pAbAi bait vector and pGADT7 prey plasmid, respectively. The bait plasmids were digested with *Bbs*I (NEB) and transformed into *Saccharomyces cerevisiae* Y1HGold. Transformed colonies were selected on synthetic dropout glucose medium (SD) without uracil (SSD). The pGADT7-MYC2 prey plasmid was introduced into the bait-integrated Y1HGold, and transformed colonies were selected on SSD media without leucine. To test the interaction between MYC2 and the *AHP6* promoter, transformed yeast lines (OD_280_ = 0.1) were dropped on SSD-Leu media containing 200 ng/ml aureobasidin A (Abs A). The dropped cells were grown at 30 °C for 4 days.

### Chromatin immunoprecipitation assay

To investigate the direct interaction between MYC2 and the *AHP6* promoter, ChIP assays were performed using *35S::MYC2-GFP* transgenic plants^54^. Around 0.5 g of roots of 7-day-old *35S::MYC2-GFP* seedlings were harvested and then immediately immersed in fixing solution (0.4 M sucrose, 10 mM Tris-HCl (pH 8.0), 5 mM β-mercaptoethanol, and 1% formaldehyde) for 15 min. After washing, the roots were ground in liquid nitrogen and nuclei were isolated. For fragmentation, the nuclei were sonicated (Ultrasonic Processor, GE-50). Immunoprecipitation was performed as described by Gendrel *et al.* except that a GFP antibody (ab290, ABcam) was used^59^. For qPCR analysis, 18S rRNA was used as an internal control. The *JAZ1* promoter was used for a positive control of the MYC2 interaction. Primer sequences are listed in Supplementary Table S2.

### Microscopy

For the visualization of root vascular tissues, whole roots of 7-day-old Arabidopsis seedlings were dipped in propidium iodide (PI) solution (10 µg/ml) for 1 min. After staining, the roots were mounted on glass slides in ddH_2_O. For the detection of PI and green fluorescent protein (GFP), fluorescence was visualized with wavelengths of 591–635 nm for PI and 505–530 nm for GFP, using a Leica SP8 STED laser scanning confocal microscope. Photographs of plants and tissue sections were taken with a Nikon SMZ-U stereomicroscope and an Olympus BX41 light microscope.

### RNA sequencing analysis

Col-0 plants were grown in 1/2× MS solid media containing 10 μM MeJA, 50 nM BAP, or 10 μM MeJA plus 50 nM BAP for 7 days. Total RNA was extracted from the roots of these plants together with those of Col-0 plants grown in 1/2x MS solid media. To generate cDNA libraries with the TruSeq RNA library kit, 1 μg of total RNA was used. Library construction consisted of polyA-selection of RNA, RNA fragmentation, random hexamer primed reverse transcription and 100 nt paired-end sequencing with the Illumina HiSeq. 2000. The libraries were quantified using qPCR according to the qPCR Quantification Protocol Guide and qualified using an Agilent Technologies 2100 Bioanalyzer. Raw data were calculated as Fragments Per Kilobase of exon model per Million mapped fragments (FPKM) of each transcript in each sample by Cufflinks software. The transcripts with zero FPKM values were removed from each data set. A combined data set of 22,291 genes was obtained by merging the data sets for each condition. For generation of the heat map of hierarchical clustering to visualize expression patterns of differentially expressed transcripts, 4,401 genes satisfying |fold change| ≥ 2 in at least one data set were collected. The GEO accession number of these datasets is GSE80188 (http://www.ncbi.nlm.nih.gov/geo/query/acc.cgi?acc=GSE80188).

### Accession numbers

Sequence data from this article can be found in the Arabidopsis Genome Initiative or GenBank/EMBL databases under the following accession numbers: *WOL* (At2G01830), *ARR6* (At5G62920), *ARR7* (At1G19050), *ARR15* (At1G74890), *AHP6* (At1G80100), *LOX2* (At3G45140), *JR2* (At4G23600), *MYC2* (At1G32640), and *GAPDH* (At1G13440).

## Electronic supplementary material

Supplementary Information
