# Supplementary material for: Antagonistic interaction between jasmonic acid and cytokinin in xylem development
Source: Sci Rep. 2017 Aug 31;7:10212. doi: 10.1038/s41598-017-10634-1 (PMC5579306; doi:10.1038/s41598-017-10634-1)
Supplement: Supplementary file 1 — Supplementary Information [file 41598_2017_10634_MOESM1_ESM.pdf]

Supplementary Information

Antagonistic interaction between jasmonic acid and cytokinin in xylem development

Geupil Jang[1], Sun Hyun Chang[1], Tae Young Um[1], Sangyool Lee[1], Ju-Kon Kim[2] and Yang Do Choi[1,*]

[1]Department of Agricultural Biotechnology and Research Institute of Agriculture and Life Sciences, Seoul National University, Seoul 151-921, Korea
[2]Graduate School of International Agricultural Technology and Crop Biotechnology Institute/Green BioScience and Technology, Seoul National University, Pyeongchang 232-916, Korea

Author for Correspondence:
Yang Do Choi

Department of Agricultural Biotechnology and Research Institute of Agriculture and Life Sciences, Seoul National University, Seoul 151-921, Korea
Email: choiyngd@snu.ac.kr

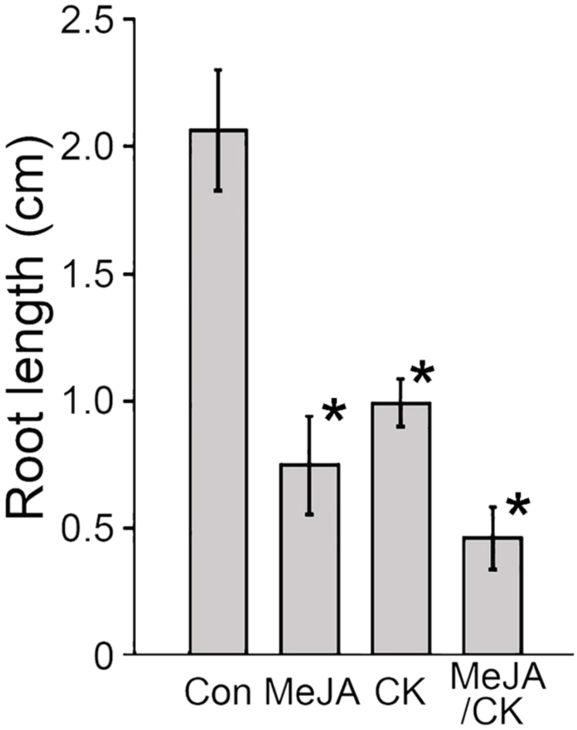

Figure S1. Inhibition of apical root growth by JA and cytokinin.

Root growth of Col-0 grown in the indicated conditions for 7 days (MeJA; 10 μM, cytokinin; 50 nM BAP, MeJA/cytokinin; 10 μM MeJA and 50 nM BAP) (*n*>20). Error bars represent S.D and asterisks indicate statistically significant differences between the corresponding samples and their control ($p < 0.01$, *t*-test).

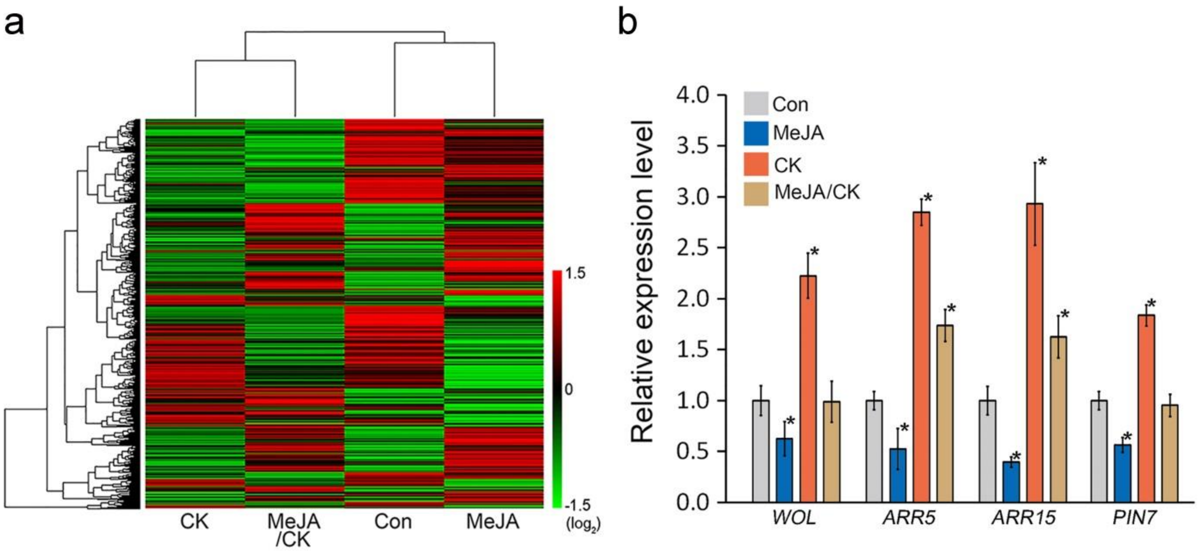

Figure S2. Antagonistic interaction between JA and cytokinin.

(**a**) Heat map showing distinct gene expression profiles between the indicated datasets. (**b**) Quantitative RT-PCR analysis of expression of *WOL*, *ARRs*, and *PIN7* in the Col-0 roots grown in the indicated conditions for 7 days (MeJA; 10 μM, cytokinin; 50 nM BAP, MeJA/cytokinin; 10 μM MeJA and 50 nM BAP). *GAPDH* was used as a reference gene. Error bars represent S.D and asterisks indicate statistically significant differences between the corresponding samples and their control ($p < 0.01$, *t*-test).

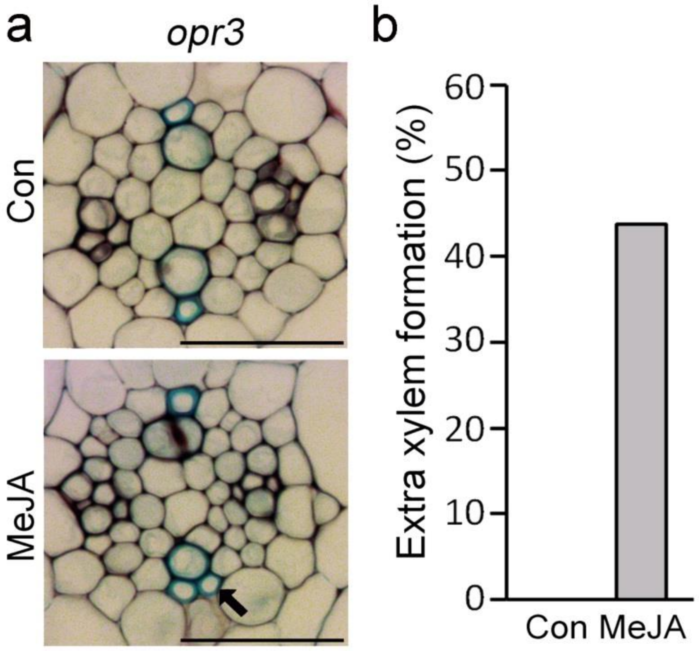

Figure S3. Formation of extra xylem in the *opr3* mutant.

(**a**) Root vasculature of *opr3* mutant plants grown in 10 μM MeJA or not treated for 7 days. (**b**) Quantification of extra xylem formation in these plants (n>15). Percentages were calculated by dividing the number of plants with extra xylem by the number of plants observed. Arrow indicates extra xylem. Scale bar = 20 μm.

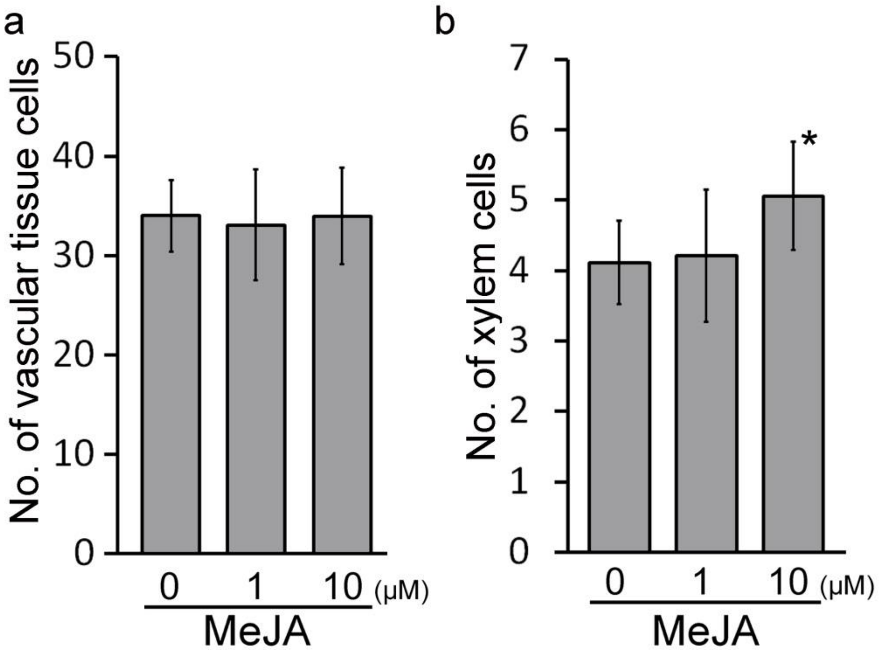

Figure S4. JA promotes xylem differentiation.

Quantification of the number of vascular tissue cells (**a**) and xylem cells (**b**) in the Col-0 roots grown in 1, 10 μM MeJA or not treated for 7 days (*n*>30). Error bars represent S.D and asterisks indicate statistically significant differences between the corresponding samples and their control ($p < 0.01$, *t*-test).

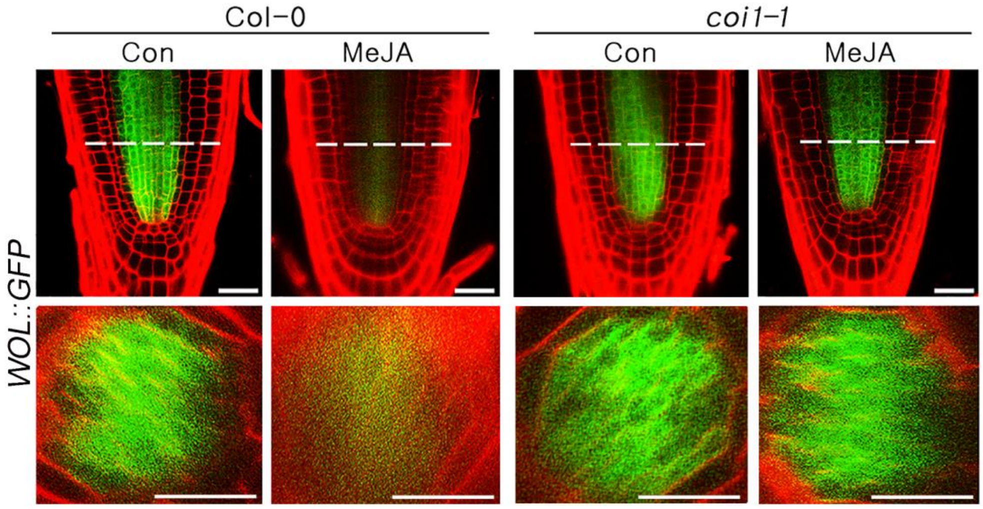

Figure S5. JA reduces cytokinin response.

Confocal longitudinal (top) and cross (bottom) sectioning images showing that MeJA reduced cytokinin-responsive *WOL* expression in the root of Col-0, but not in *coi1-1* mutants. Dotted lines indicate the longitudinal position where confocal optical cross-sectioning was performed. Scale bar = 20 μm.

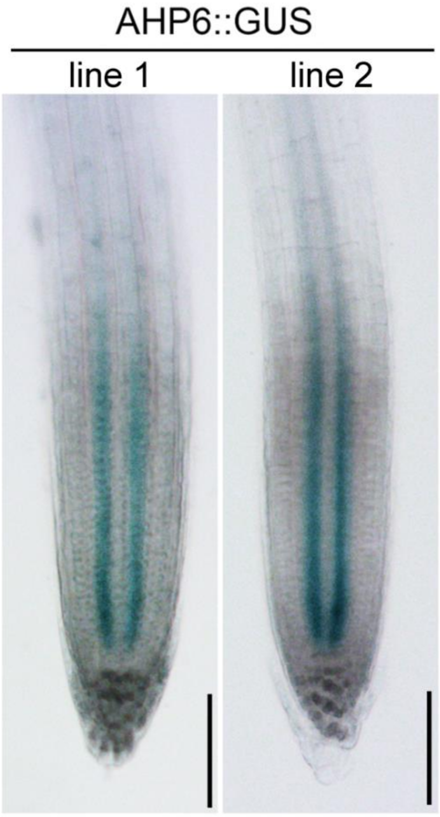

Figure S6. GUS staining analysis of *AHP6::GUS* plants.
GUS staining of two independent lines of *AHP6::GUS* plants grown in MS media for 7 days. Scale bars = 100 μm.

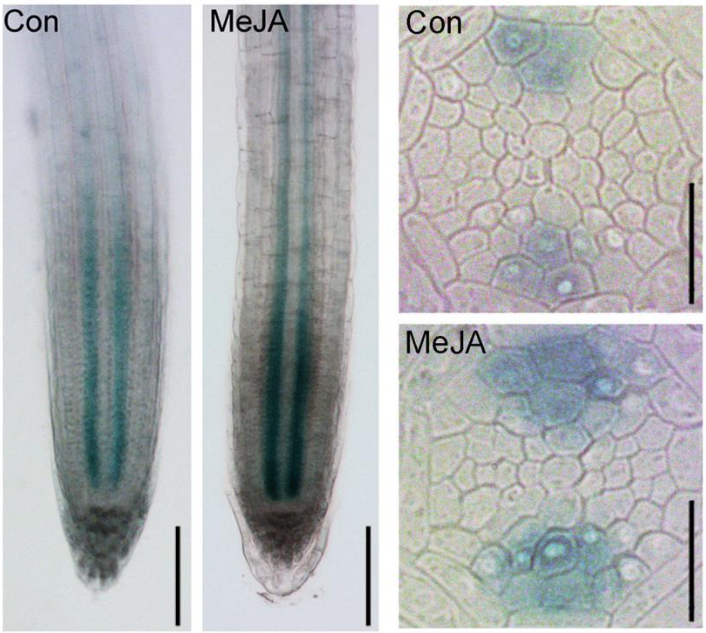

Figure S7. JA increases expression of *AHP6*.

Analysis of *AHP6* expression patterns in Arabidopsis roots grown in 10 μMMeJA-untreated and -treated conditions using GUS staining. GUS staining solution with 1 mM ferrocyanide/ferricyanide was used. Whole mounted (left), and cross-sectioning images (right). Scale bar = 100 μm in whole mounted images and 20 μm in others.

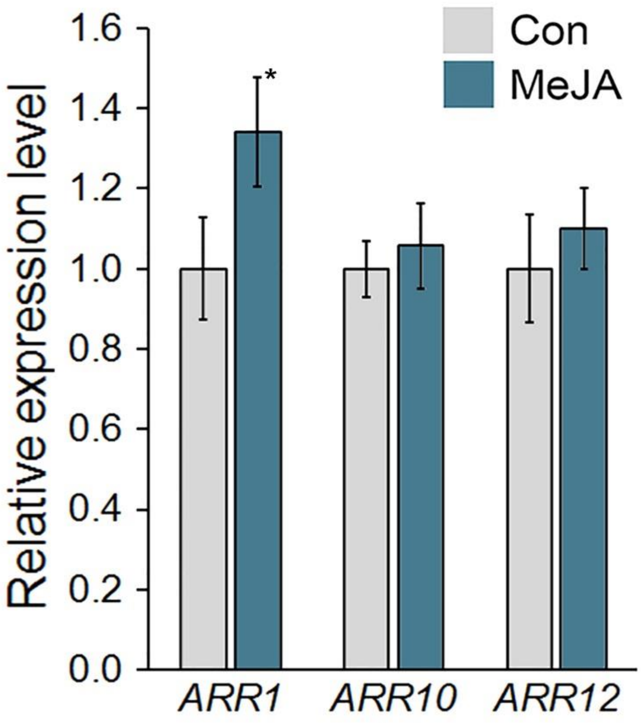

Figure S8. Expressions of Type-B *ARRs* in JA-treated Col-0 plants.

Quantitative RT-PCR analysis of expression of Type-B *ARRs* in the Col-0 roots grown in 10 µM MeJA-untreated and -treated conditions for 7 days. *GAPDH* was used as a reference gene. Error bars represent S.D and asterisks indicate statistically significant differences between the corresponding samples and their control (p < 0.01, t-test).

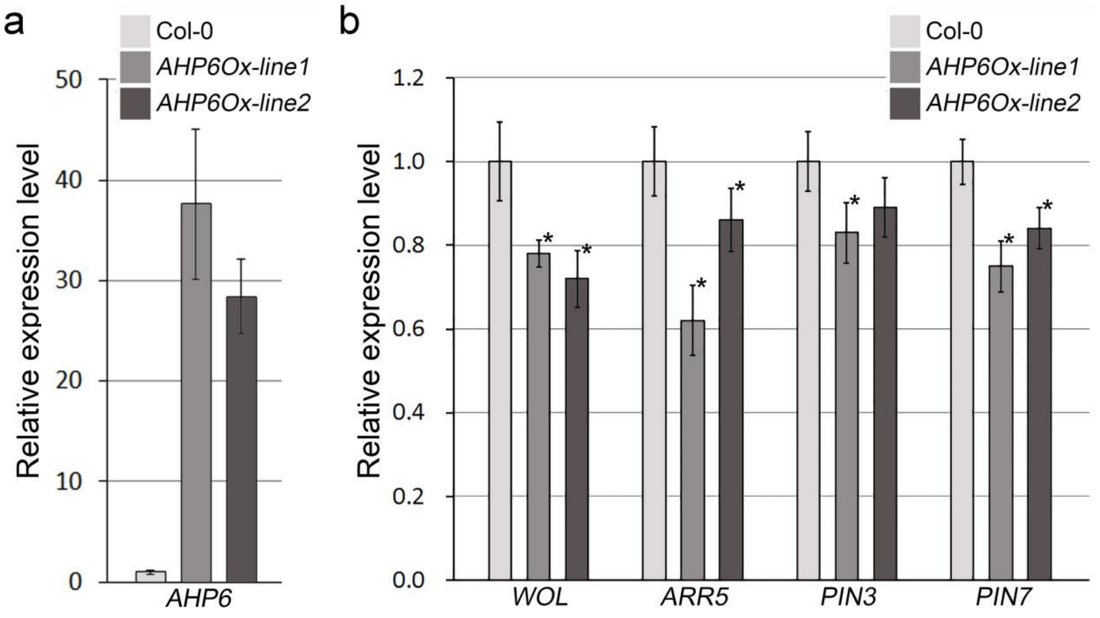

Figure S9. Reduced cytokinin response in *35S::AHP6* plants.

Expression levels of *AHP6* (**a**) and cytokinin-induced genes (**b**) in the roots of *AHP6*-overexpressing transgenic plants grown in MS media for 7 days. Line 1 and 2 indicate individual lines of *AHP6*-overexpressing transgenic plants. *GAPDH* was used as a reference gene. Error bars represent S.D and asterisks indicate statistically significant differences between the corresponding samples and their control ($p < 0.01$, *t*-test).

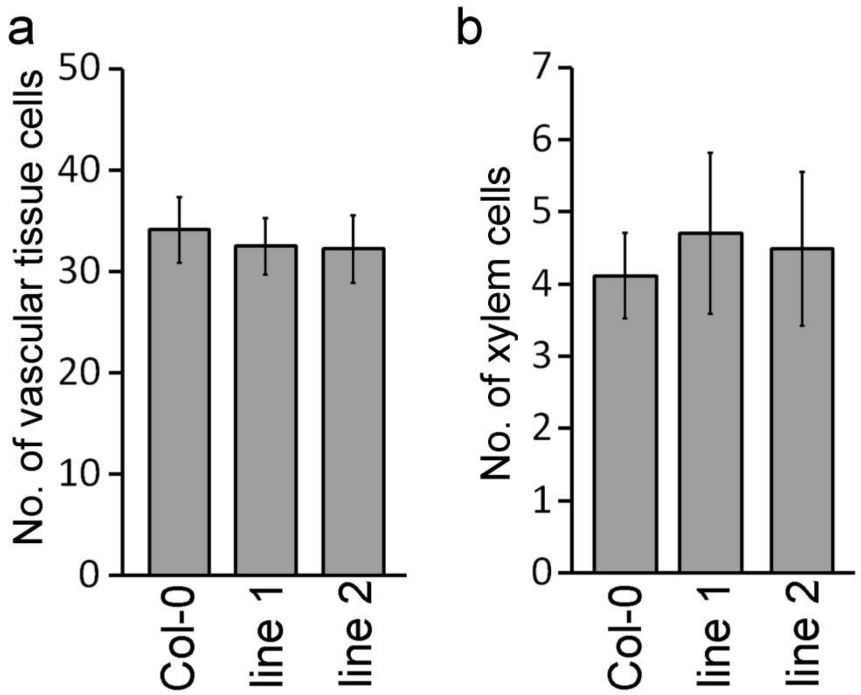

Figure S10. Overexpression *AHP6* tends to increase the number of xylem cells. Quantification of the number of vascular tissue cells (**a**) and xylem cells (**b**) in the roots of *35S::AHP6* plants roots grown in JA-untreated conditions for 7 days (*n*>30). Error bars represent S.D.

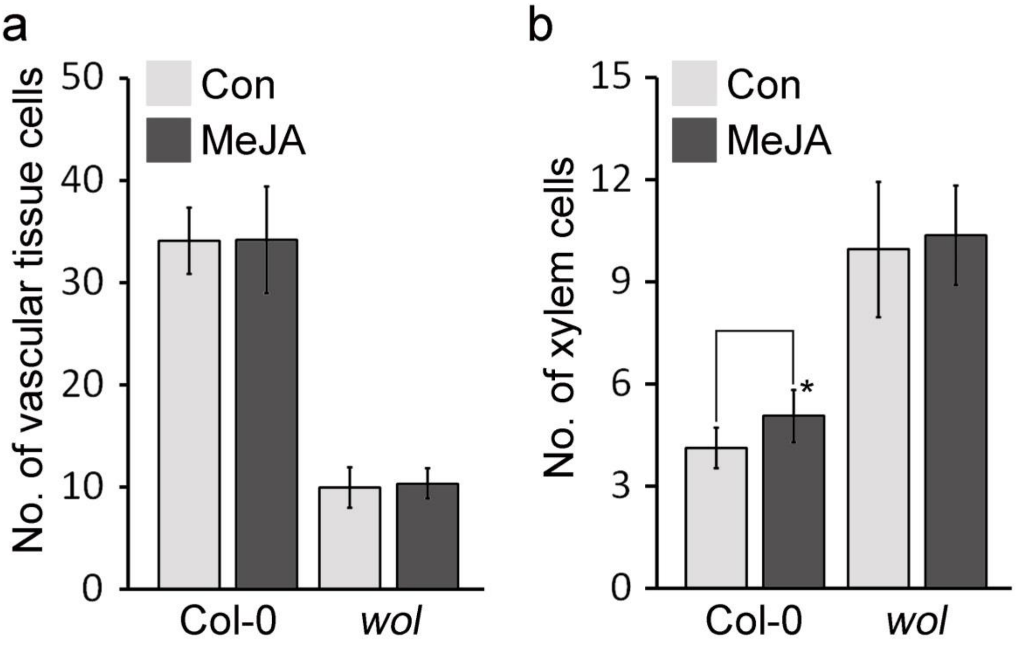

Figure S11. JA does not affect xylem development in *wol* mutant plants.

Quantification of the number of vascular tissue cells (**a**) and xylem cells (**b**) in the roots of *wol* mutant plants grown in 10 μM MeJA or not treated for 7 days (n>13). Error bars represent S.D and asterisks indicate statistically significant differences between the corresponding samples and their control ($p < 0.01$, t-test).

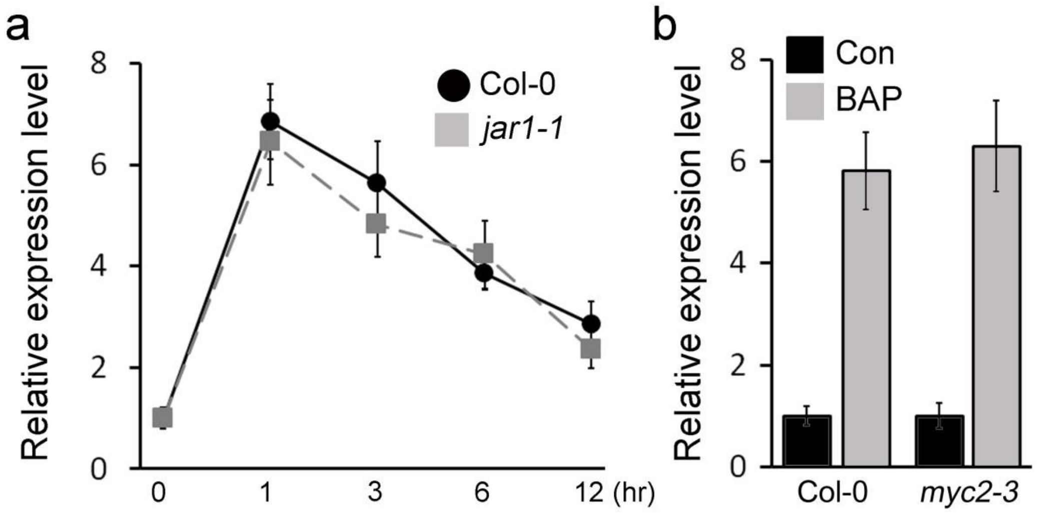

Figure S12. Cytokinin responses in JA-signaling defective mutants.

(**a**) *ARR5* expression was analyzed in *jar1-1* plants by qRT-PCR. Seven-day-old *jar1-1* and Col-0 seedlings were transferred to MS media containing 2 μM BAP and incubated for the indicated time. Total RNA was extracted from these roots. (**b**) Expression levels of *ARR5* in the roots of *myc2-3* mutants treated or untreated with 2 μM BAP for 3 hrs. *GAPDH* was used as a reference gene. Error bars represent S.D.

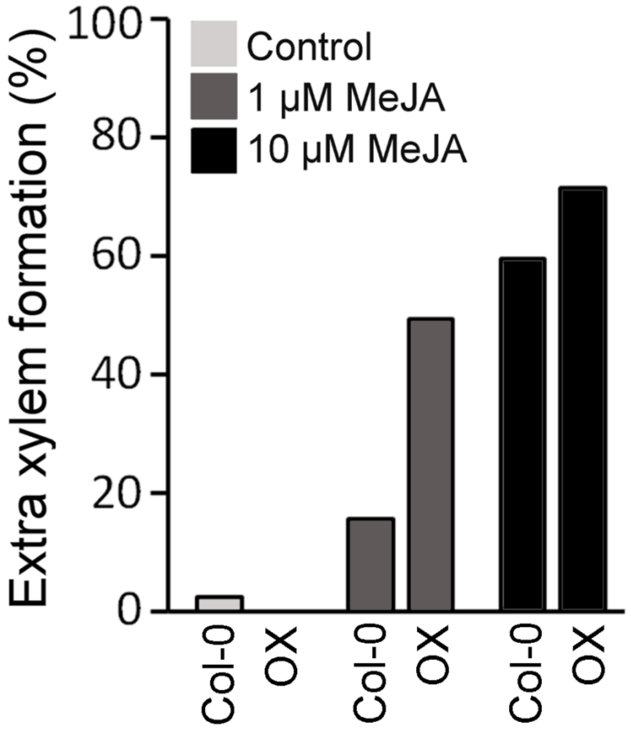

Figure S13. MYC2 promotes formation of extra xylem in response to JA. Quantification of extra xylem formation in the roots of Col-0 and *35S::MYC2-GFP* (OX) grown in 1 and 10 µM MeJA-treated and -untreated conditions for 7 days (*n*>20). Percentages were calculated by dividing the number of plants with extra xylem by the number of plants observed.

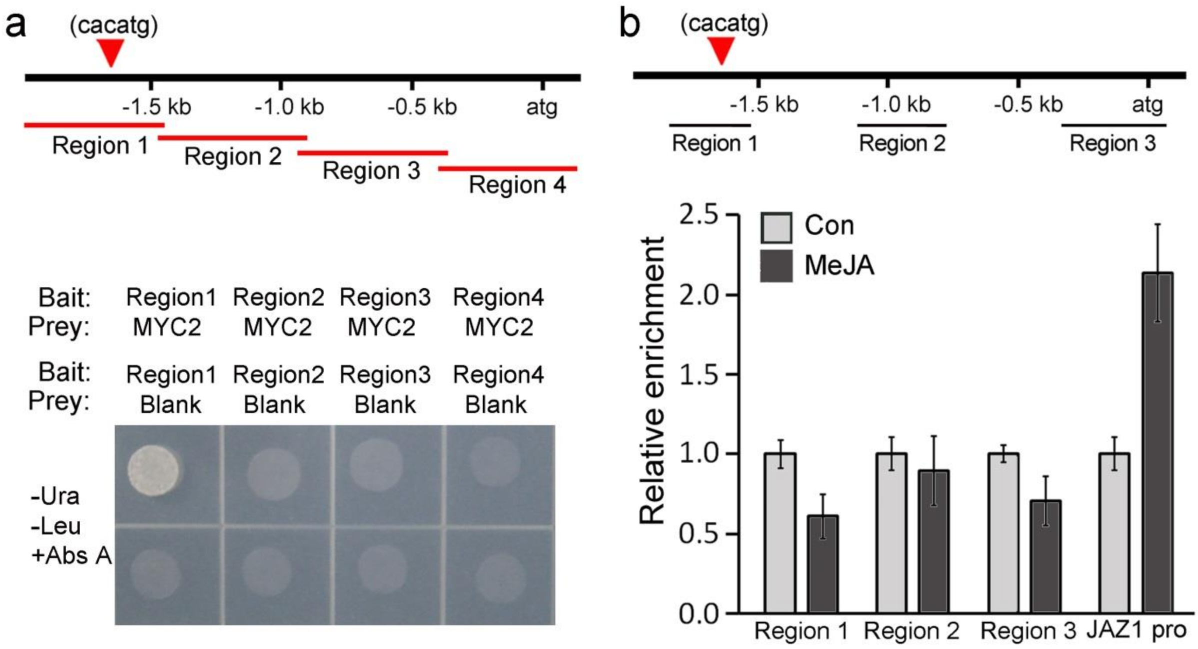

Figure S14. MYC2 does not bind to the *AHP6* promoter.

(**a**) Schematic of the *AHP6* promoter containing a putative MYC2-binding site (CACATG), and yeast one-hybrid results. Regions 1, 2, 3, and 4 indicate DNA fragments of the *AHP6* promoter inserted into the pAbAi bait plasmid. A full-length *MYC2* cDNA was inserted into the pGADT7 prey plasmid. -Ura -Leu + Abs A (150 μM aureobasidin A) media were used to test the interaction between the the *AHP6* promoter and MYC2. (**b**) Chromatin immunoprecipitation (ChIP) assay using plants expressing *35S::MYC2-GFP* and GFP antibody. Plants were treated with MeJA for 4 hrs by transferring 7-day-old *35S::MYC2-GFP* seedlings into MS media containing 100 μM MeJA. Enrichment of the indicated regions was analyzed by qPCR. *18S rRNA* was used as a reference gene. Error bars represent S.D.

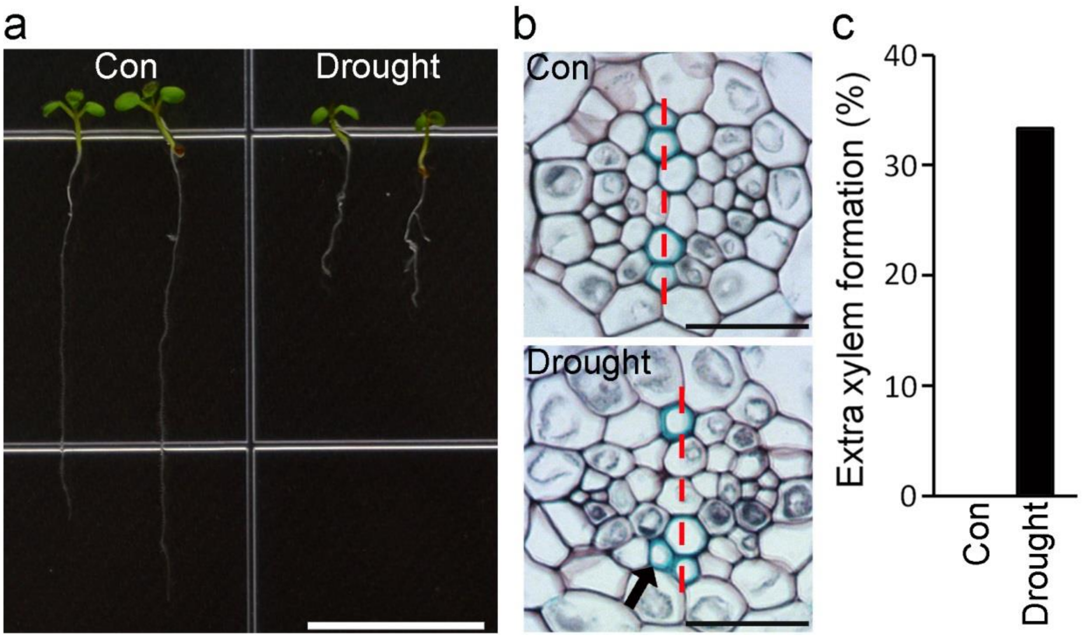

Figure S15. Drought induces extra xylem cells.

Root growth (**a**) vascular morphology (**b**) of Col-0 plants grown PEG-infused MS media. Four-day-old seedlings germinated on MS media were transferred to the control media (1/2 MS medium (Water potential = -0.25 MPa) and PEG-containing media (Water potential = -0.70 MPa), respectively. After 8 days, roots were collected from these plants and sectioned transversly. The red dotted line indicates a xylem axis. Arrow indicates an extra xylem (**c**) Quantification of extra xylem formation in these plants (*n*>15). Percentages were calculated by dividing the number of plants with extra xylem by the number of plants observed. Scale bar = 1 cm in (a) and 20 μm in (b).

Table S1. **Putative MYC2-binding sites in the *AHP6* promoter.**

Locus Name	Position	Strand	Sequence	Silmilar Score
AT1G32640	-1623	+	CACATg	1
AT1G32640	-1482	+	ATACGaat	0.75
AT1G32640	-1482	-	ataCGAAT	0.75
AT1G32640	-1342	-	aaaAGTAT	0.75
AT1G32640	-1336	+	ATATGttt	0.75
AT1G32640	-1079	+	ATACGtgc	0.88
AT1G32640	-987	-	tcaCGTAA	0.75
AT1G32640	-963	+	ATACAttt	0.75
AT1G32640	-669	+	ATACGagg	0.75
AT1G32640	-384	-	agaTGTAT	0.75
AT1G32640	-215	+	ATACTtgt	0.88
AT1G32640	-53	+	ATACCtct	0.75

Table S2. **Primers used in this study.**

Name	Sequence
ARR1 RT 5 for	CACATCAGGACTCAGAATCC
ARR1 RT 3 rev	CGTCTGCATAAACCTGATG
ARR10 RT 5 for	GGGATTTGGATGGTTTAGCTC
ARR10 RT 3 rev	CAGAGAAGAGAGAAGAGACAG
ARR12 RT 5 for	GGAGGAGATTTGGGATATGG
ARR12 RT 3 rev	CTAAATTGCTAGAACCAGTGGG
ARR5 RT 5 for	GCACAAGAGAGAGCTTGAAGC
ARR5 RT 3 rev	TCAGGACATGCATGTGTGTG
ARR6 RT 5 for	GTTCACTCGCAGCTCAAAC
ARR6 RT 3 rev	GGCGAGAATCATCAGTGTAG
ARR7 RT 5 for	CCTCTTCTTGGAACCAATCTG
ARR7 RT 3 rev	CATCGACGGCAAGAACATG
ARR15 RT 5 for	GAGAGGTGGTGAAGCTGAAG
ARR15 RT 3 rev	GTCGTCATCAAGGGAGGAAAC
WOL RT 5 for	GCCGAGAAAGATCGATTTTTGG
WOL RT 3 rev	GCTTTTGGTAACAATGCTCGATG
PIN3 RT 5 for	GCCCTTTGTGTTTGCGAAG
PIN3 RT 3 rev	CAATTGACTCTTTCTCGGGG
PIN7 RT 5 for	CATCCCGCAATCTTGAGTAC
PIN7 RT 3 rev	CCTCTTCAGCCAAGCAGAAC
AHP6 RT 5 for	GTGCTTGAGAGGACTGGAGG
AHP6 RT 3 rev	TACATTGGATATCTGACTCCTG
LOX2 RT 5 for	GAGCCTGTTATCAATGCTGCA
LOX2 RT 3 rev	ATTCCGGTAACACCATGCTCA
JR2 RT 5 for	CAGTCAGAAGAACTGGTTGAG
JR2 RT 3 rev	AGGCAAATAACCATATTGACCC
ACT2 RT 5 for	CTTGCACCAAGCAGCATGAA
ACT2 RT 5 for	CCGATCCAGACACTGTACTTCC
GAPDH RT 5 for	GCATTGAGCGACAAGTTTGTG
GAPDH RT 3 rev	AGTACGAACTCAACCACACAC
ChIP R1 5 for	GTCAAAGCAAAGGAATGACGAA
ChIP R1 3 rev	TGTCTCATTCGTATGTCATGC
ChIP R2 5 for	GCGATGTGATACCAATATACCA
ChIP R2 3 rev	GGCCTTTCTCTTTCTCGATCTA
ChIP R3 5 for	GGGGGAAAAAGGTAACCTGAC
ChIP R3 3 rev	TCGGCTTGAAGCCGGTCCAC

ChIP JAZ1 5 for	TTCAAAGAAACGGAAAGC
ChIP JAZ1 3 rev	CTACCTCCAATATAACAAAGC
18 rRNA 5 for	TACCGTCCTAGTCTCAACCA
18 rRNA 3 rev	AACATCTAAGGGCATCACAG
Bait R1 5 for	ATAAAGCTTATCTCAATGACTCAT
Bait R1 3 rev	ATAGTCGACGGAACAAACAACTAG
Bait R2 5 for	ATAAAGCTTTACTACTTACTCTGA
Bait R2 3 rev	ATAGTCGACCGAATAAAGTAAAAA
Bait R3 5 for	ATAAAGCTTATAGTTAAGAGGACT
Bait R3 3 rev	ATAGTCGACTTCTTTTATCAGGTC
Bait R4 5 for	ATAAAGCTTTGTTATATGATTATA
Bait R4 3 rev	ATAGTCGACCCACAACGGCACACC
Prey MYC2 5 for	ATACATATGATGACTGATTACCGG
Prey MYC2 3 rev	ATAATCGATTTAACCGATTTTTGA
AHP6 cDNA 5 for	GGGGACAAGTTTGTACAAAAAAGCAGGCTCCATGTTGGGGTTGGGTGTGGA
AHP6 cDNA 3 rev	GGGGACCACTTTGTACAAGAAAGCTGGGTCTTACATTGGATATCTGACTCCTG
AHP6 pro 5 for	TACGCCAAGCTTGGCTGCAGATCTCAATGACTCATCATATC
AHP6 pro 3 rev	AAAACGACGGCCAGTGAATTCCCACAACGGCACACCCGT
